# Integrated genomic and proteomic profiling reveals insights into chemoradiation resistance in cervical cancer

**DOI:** 10.1002/1878-0261.70108

**Published:** 2026-01-26

**Authors:** Janani Sambath, Irene A. George, Srikanth S. Manda, Prasanth Ariyannur, Ekta R. Dhawale, Raja Sekhar Kommu, Rajan Datar, Darshana Patil, Vinita Trivedi, Manisha Singh, Kumar Prabhash, Sewanti Limaye, Richa Chauhan, Prashant Kumar

**Affiliations:** ^1^ Manipal Academy of Higher Education (MAHE) India; ^2^ Institute of Bioinformatics International Technology Park Bangalore India; ^3^ Nucleome Informatics Pvt Ltd, HITEC City Hyderabad India; ^4^ Karuna Medical College Palakkad India; ^5^ Vedantaa Hospital & Research Centre Palghar India; ^6^ Karkinos Healthcare Pvt Ltd Mumbai India; ^7^ Datar Cancer Genetics Nashik India; ^8^ Mahavir Cancer Sansthan Patna India; ^9^ Tata Memorial Hospital Mumbai India; ^10^ Homi Bhabha National Institute Mumbai India; ^11^ Sir H N Reliance Foundation Hospital and Research Centre Mumbai India; ^12^ Indian Institute of Technology Kanpur India

**Keywords:** biomarker, cervical cancer, chemoradiation resistant, proteomics, whole‐genome sequencing

## Abstract

Cervical cancer is highly prevalent in India, with most cases being diagnosed at advanced stages. Despite the standard concurrent chemoradiotherapy (CCRT), 30–40% of patients' experience treatment failure, underscoring the need for improved therapeutic strategies. Understanding resistance mechanisms and identifying predictive biomarkers are crucial to improve treatment efficacy and enable personalized medicine. We conducted a comprehensive genomic and proteomic analysis to identify molecular signatures associated with CCRT. We identified recurrent mutations in phosphatidylinositol 4,5‐bisphosphate 3‐kinase catalytic subunit alpha isoform (*PIK3CA*) and histone‐lysine N‐methyltransferase 2D (*KMT2D*), with mutation signature analysis revealing a prevalent DNA dC‐ > dU‐editing enzyme, APOBEC mutagenesis signature. Distinct genomic alterations, including epidermal growth factor receptor (*EGFR*) amplification and serine/threonine kinase 11 (*STK11*) deletion, were exclusively observed in the chemoradiation‐resistant cohort. Proteomic analysis identified 73 significantly dysregulated proteins, with syntaxin‐3 (STX3), SERPINB7, lipopolysaccharide‐binding protein (LBP), EMILIN2, and ribosyldihydronicotinamide dehydrogenase (quinone) (NQO2) being the top five upregulated proteins. Integrative pathway analysis highlighted an active DNA repair pathway in the resistant cohort. This study presents the first proteogenomic profiling of cervical cancer in the Indian population, linking molecular alterations to CCRT response. *STK11* and STX3 emerged as predictive biomarkers for poor response, whereas *EGFR* presents as a promising therapeutic target in the resistant group.

## Graphical abstract

A comprehensive genomic and proteomic analysis of cervical cancer revealed STK11 and STX3 as a potential biomarkers of chemoradiation resistance. Our study demonstrated EGFR as a therapeutic target, paving the way for precision strategies to overcome treatment failure and the DNA repair pathway as a critical mechanism of resistance.

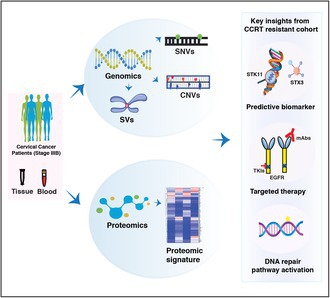

**Abbreviations**
APOBEC: apolipoprotein B mRNA editing catalytic polypeptide‐like
CCRT: concurrent chemoradiation therapy
CGC: cancer gene census
CGR: complex genomic rearrangement
CNA: copy number alteration
COSMIC: catalog of somatic mutations in cancer
EGFR: epidermal growth factor receptor
FIGO: International Federation of Gynecology and Obstetrics Classification
HPV: human papillomavirus
LACC: locally advanced cervical cancer
LC–MS/MS: liquid chromatography–tandem mass spectrometry
PFI: progression‐free interval
RECIST: response evaluation criteria in solid tumors
SNV: single nucleotide variant
STK11: serine/threonine kinase 11
STX3: syntaxin 3
SV: structural variants
TCGA: the cancer genome atlas
TMB: tumor mutation burden
TMT: tandem mass tag
WES: whole‐exome sequencing
WGS: whole‐genome sequencing

## 1 Introduction

Cervical cancer ranks as the fourth most common cancer in women globally and is a leading cause of gynecological cancer mortality, particularly in low‐ and middle‐income countries [1]. In India, cervical cancer is the second most prevalent malignancy among women, contributing to 10% of all female cancer cases, following breast cancer in incidence [2]. Persistent infection with high‐risk human papillomavirus (HPV) subtypes, particularly types 16 and 18, is the primary cause of cervical cancer [3]. Despite the availability of effective screening and vaccination programs, approximately 80% of the cases in India are diagnosed at locally advanced stages [4, 5]. Cisplatin‐based concurrent chemoradiotherapy (CCRT), which combines external beam radiotherapy and intracavitary brachytherapy with concurrent cisplatin, is the standard treatment for locally advanced cervical cancer (LACC), reducing mortality risk by 30–35% [6]. However, 30–50% of patients with LACC experience treatment failure leading to tumor recurrence and distant metastasis, with 5‐year survival rates remaining below 60% [7, 8]. Enhancing long‐term survival, preventing treatment resistance, and minimizing metastasis in LACC remains a significant challenge. Despite numerous studies and ongoing trials exploring new therapeutic strategies and combinations, including the integration of targeted therapies like bevacizumab and pembrolizumab, improvements in response rates and overall survival remain modest, highlighting the need for innovative approaches [9, 10, 11].

A deeper understanding of disease mechanisms and resistance pathways is crucial for developing effective treatments and identifying new targets to improve disease management. Large‐scale genomic studies, including the TCGA effort, identified recurrent mutations in genes, such as *PIK3CA*, *FBXW7*, *EP300*, *PTEN*, *HRAS*, *HLA‐B*, and an APOBEC‐related mutagenesis pattern [12, 13]. Prior studies have linked mutations, such as *BRCA1* and *PIK3CA*‐*E545K* to CCRT resistance in cervical cancer, by activating the JAK/STAT and PI3K/Akt/mTOR pathways, respectively [14, 15]. While these *in vitro* studies have shed insights into therapy resistance, the comprehensive investigation of the molecular mechanisms driving resistance is not systematically explored. The heterogeneity in treatment response necessitates personalized profiling of each patient to enable biomarker‐driven therapy selection, optimizing treatment efficacy while avoiding overtreatment and cost [16]. Independent studies have shown measures of hypoxia, excision repair cross‐complementation group 1 enzyme expression, and neutrophil‐lymphocyte ratio could correlate with the differential response of the patients toward CCRT, but these markers alone are insufficient to predict the treatment response [17]. Therefore, there is an urgent need to discover novel predictive biomarkers that can guide treatment decisions, avoiding toxicity associated with the treatment and improving the toxicity to benefit ratio.

In an effort to elucidate the mechanisms of therapy resistance in cervical cancer and to identify novel predictive biomarkers, we conducted an integrative analysis using whole‐genome sequencing (WGS) and high‐resolution mass spectrometry. We characterized the first genomic landscape of cervical cancer within the Indian population using WGS, offering crucial insights into region‐specific genomic alterations. Furthermore, we identified distinct genomic and proteomic signatures between chemoradiation‐resistant and sensitive patients, uncovering key biological differences linked to CCRT resistance. These findings not only enhance our understanding of resistance mechanisms but also present promising biomarkers for improving patient stratification and optimizing therapeutic strategies.

## 2 Materials and methods

### 2.1 Sample acquisition and clinical data

Tumor tissue and matched blood samples were collected from newly diagnosed stage IIIB cervical cancer patients through biopsy during the period of September 2021–December 2022. After the biopsy, all the patients underwent concurrent chemoradiation therapy (CCRT) at Mahavir Cancer Sansthan, Patna. The stage classification of the cervical cancer cases was done using the FIGO (International Federation of Gynecology & Obstetrics) system. The inclusion criteria for this study were defined to include only patients with Stage IIIB squamous cell carcinoma who were HPV‐positive, in order to minimize confounding effects related to histological heterogeneity and HPV status, both of which are known to influence treatment response and molecular profiles. Thirty‐six tumor tissues were collected, in which only 25 samples had matched blood samples. All the patients were monitored after the treatment and based on the RECIST (Response Evolution Criteria in Solid Tumors) criteria, the patients are classified as sensitive and resistant cohort [18]. Out of 36 samples, 19 samples were classified as the chemoradiation‐sensitive group who showed complete response, and 17 were classified as the chemoradiation‐resistant group who showed stable disease or progressive disease (Table S1).

### 2.2 Ethics declaration

The samples were collected from patients enrolled in Mahavir Cancer Sansthan, Patna, Bihar, India. Written informed consent was obtained from all patients, and the Institutional Ethics Committee of Mahavir Cancer Sansthan has approved the project (MCS/IEC No.: 01/05/2018/413). The study was conducted in accordance with the national ethical guidelines for biomedical and health research involving human participants (Indian Council of Medical Research, India) and with the principles outlined in the Declaration of Helsinki and its subsequent amendments.

### 2.3 Protein extraction, LC–MS/MS and data analysis

Protein extraction was carried out from the tissue samples, and 100 μg of protein from each sample was reduced with 10 mm DTT, alkylated with 20 mm iodoacetamide, and precipitated using ice‐cold acetone. The precipitate was resuspended in 50 mm TEABC and digested with trypsin (1 : 20) at 37 °C for 12–16 h. The resulting peptides were cleaned with Sep‐Pak C18 material, labeled with 10‐plex TMT reagents, pooled, and separated via basic pH reverse‐phase liquid chromatography (bRPLC) on a C18 column, yielding 12 fractions for LC–MS/MS analysis. Peptide fractions were analyzed using a Q Exactive HF‐X Orbitrap mass spectrometer coupled to a Dionex Ultimate 3000 system. Peptides were separated on a C18 analytical column using a 120‐min gradient. Data acquisition was performed in a data‐dependent mode with precursor scans (350–1600 m/z) at a resolution of 120 000, and fragment scans acquired at 45 000 resolutions following HCD fragmentation. Data were processed using the SEQUEST search algorithm in Proteome Discoverer (v2.1) against the Human RefSeq protein database. Search parameters included a 10 ppm precursor tolerance, 0.02 Da fragment tolerance, and modifications for TMT labeling, carbamidomethylation, and variable oxidation and deamination. Results were filtered at a 1% FDR using a decoy database. The workflow followed for the proteomics analysis is depicted in Fig. S1. For identifying the differentially expressed proteins between the nonresponders and responders, two‐sample ‘*t*‐test’ with equal variance was carried out (*P* < 0.05).

### 2.4 DNA extraction and whole‐genome sequencing

Genomic DNA was extracted from cancer tissues using the QIAamp Fast DNA tissue kit, and matched blood DNA was extracted from peripheral blood using the QIAamp DNA Blood Mini Kit (Qiagen, Hilden, Germany) as per standard protocols. DNA concentrations will be evaluated using a Nanodrop spectrophotometer (Thermo Scientific, Waltham, MA, USA), Qubit Fluorometer (Thermo Scientific) and Tape Station fragment analyzer (Agilent, Santa Clara, CA, USA). After quantification and quality control, 100–300 ng of genomic DNA was used with a LE220‐plus Covaris Focused‐ultrasonicator (Covaris, Woburn, MA, USA; catalog # 500569) and sequencing libraries were prepared using the Illumina DNA PCR‐Free Prep (Illumina, San Diego, CA, USA; Catalog# 20041795) as per standard protocol. Library quantification was performed using the Kappa NGS library quantification kit (Roche, Mannheim, Germany; catalog # KK4824) and PCR‐free libraries were pooled for sequencing. The DNA library with 150‐bp paired‐end reads was sequenced with the Illumina NovaSeq 6000 at a mean depth of around 33×. WGS was performed on a total of 26 tumor samples, including 15 paired tumor–blood samples and 10 unpaired tumor samples without matched normal DNA.

### 2.5 Whole‐exome sequencing

DNA was extracted from tumor samples and peripheral blood using the QIAamp DNA tissue/blood Kit. The DNA quantity was evaluated using both a Nanodrop spectrophotometer and a Qubit fluorometer. To assess the integrity of the extracted DNA, 1% agarose gel electrophoresis was performed. A minimum of 100 ng of DNA with a 260/280 absorbance ratio greater than 1.6 was used as input for the whole‐exome library preparations using the Agilent SureSelectXT Human All Exon V5 kit. The obtained libraries were diluted to a final concentration of 2 nm in 10 μL and were subjected to cluster amplification. Once the cluster generation was completed, the flow cells were loaded onto the sequencer. Sequencing was carried out on Illumina HiSeq platform, generating 2 × 100‐bp paired‐end reads. Whole‐exome sequencing (WES) was performed on 10 paired tumor–blood samples.

Exome sequencing was used alongside WGS as a complementary and orthogonal strategy. While WGS provided a comprehensive overview of the entire genome, WES was specifically used to focus on the protein‐coding regions. Leveraging WES in conjunction with WGS improved the accuracy and robustness of genetic analyses, reduced false positives, and increased confidence in variant calls related to disease‐associated genes. A violin plot (Fig. S2) illustrates the distribution of uniquely mutated variants in WES, WGS, and those commonly detected, along with their depth of coverage, further emphasizing the complementary nature of these approaches.

### 2.6 Somatic mutation calling and annotation

Whole‐genome sequencing reads were subjected to a quality check, and low‐quality reads were excluded. The sequencing coverage and quality statistics for each sample are summarized in Table S2. HPV detection and genotyping were performed using the HPVDetector tool [19] in QuickDetect mode to identify HPV presence and subtype in each sample. The filtered reads were mapped to the hg38 reference sequence using BWA. PCR duplicates were removed using Picard, followed by indel realignment and base recalibration with GATK tools [http://broadinstitute.github.io/picard/]. Somatic mutations were identified using mutect2 [20], annotated with annovar [21]. For somatic mutation calling, a panel of normals was generated using the 15 matched blood samples and used as the normal for analyzing the unmatched tumor samples. After annotation, germline variants were filtered out using the 1000 Genomes Project, Exome Aggregation Consortium, and Genome Aggregation Database databases. For downstream filtering, a high‐quality variant was defined using the following criteria: a read depth of at least five supporting reads, at least two reads supporting the alternate allele in the tumor sample, and exclusion of variants identified in the dbSNP150 database and COSMIC. Additionally, only variants with a CADD score ≥ 10 were selected for further analysis.

We employed ‘dNdScv’ package to estimate the relative rates of nonsynonymous and synonymous mutations (dN/dS). A stringent statistical threshold of *q* < 0.1 was applied to identify significantly mutated genes under positive selection with high confidence [22]. The same approach was used for WES analysis, with hg19 as the reference genome. After variant calling, the variants were uplifted to the hg38 reference genome for further interpretation. tumor mutational burden (TMB) for each sample was determined by counting all somatic mutations, including base substitutions and indels, within the coding areas. To enhance the accuracy, synonymous mutations were also considered. The TMB value was then calculated by dividing the total mutation count by the coding region.

The maftools R package was utilized for further analysis, statistical calculations, and visualization of annotated variants from MAF files [23].

### 2.7 Mutational signature analysis

Mutational signature analysis was performed to elucidate the underlying mutational processes driving cervical cancer using the MutationPatterns R package (version 3.16) [24]. Somatic variant data were categorized into six base substitution types (C>A, C>G, C>T, T>A, T>C, and T>G), and a 96‐trinucleotide mutation count matrix was generated. *De novo* mutational signature was extracted using non‐negative matrix factorization to identify novel mutational processes specific to our cohort. The resulting *de novo* signatures were compared with the COSMIC cancer mutational signature database (v3.2) to assess their similarity to known mutational processes [25]. Additionally, the contribution of each COSMIC signature to the mutational landscape of individual samples was estimated to identify the most prominent mutational processes.

### 2.8 Copy number variation analysis

Copy number variations (CNVs) were identified from WGS data using a python library and command‐line software toolkit, cnvkit (v0.9.8) [26]. cnvkit was configured to run in batch WGS mode (‐‐method wgs), which analyzes the entire genome as a single target region to maximize sensitivity across diverse genomic areas. To improve the accuracy and reduce individual sample variability, a pooled reference was constructed by aggregating BAM files from multiple normal samples, which served as a control for contrasting against tumor samples. A gene annotation database was integrated into the analysis to label gene names in the output files. To identify recurrent genomic alterations with potential oncogenic significance, gistic (version 2.0.23) was employed with default parameters to detect significant focal amplifications and deletions [27]. For WES data, similar analysis principles were applied using cnvkit, with appropriate adjustments for the targeted regions.

### 2.9 Structural variant analysis

Structural variants were called using manta (v1.6.0) with default parameters on WGS data to detect somatic deletions, insertions, inversions, and translocations [28]. The identified SVs were annotated using AnnotSV to filter out potential false positives [29].

### 2.10 Pathway analysis

The pathway enrichment analysis was performed using ‘enricher’ function from the ‘clusterprofiler’ r package (default parameters) with the utilization of the 50 hallmark gene sets downloaded from MsigDB [30]. The most significantly enriched pathways had a *P*‐value ≤ 0.05 and contained at least two overlaps from our dataset.

### 2.11 Detection of complex genomic rearrangements (CGRs) and their signatures

We identified complex genomic rearrangements (CGRs) from WGS data by analyzing structural variations (SVs) and CNVs. The Starfish tool was used to integrate SV and CNV signals, and results were compared against six CGR signatures deconvoluted based on event topology, which enabled the classification of rearrangements across the samples [31].

### 2.12 Identification of potentially actionable events

We identified actionable mutations from WGS and WES data using the OncoKB database [32]. Mutations were classified according to OncoKB evidence Levels 1 (FDA‐approved drugs), 2 (standard care), 3 (clinical evidence), and 4 (biological evidence), representing varying degrees of clinical actionability.

### 2.13 Immunohistochemistry

Immunohistochemistry was carried out as per the standard protocol. The tissue slides for the sensitive and resistant patients were obtained from Mahavir Cancer Sansthan, Patna. We deparaffinized the tissue section and then to retrieve the antigen we incubated the sections for 20 min in 0.01 m Trisodium citrate buffer, pH 6. Endogenous activity of peroxidase was inhibited using 1 : 1 PBS and hydrogen peroxide solution. For blocking the nonspecific binding of the antibody, we incubated the sections with 5% goat serum for 20 min. Furthermore, the sections were incubated with anti‐syntaxin 3 antibodies (ab133750) overnight at 40 °C in humidified conditions. The sections were then incubated in horseradish peroxidase conjugated rabbit secondary antibody for 30 min at room temperature followed by staining with DAB chromagen and counterstained with hematoxylin. Images were then taken at 20× on an Olympus DP‐21 microscope.

### 2.14 Proteogenomic analysis

We aimed to investigate the impact of genomic alterations on the proteome, as both the WES and proteomics samples were derived from the same patients. Specifically, we examined the cis and trans effects of cancer‐associated genes harboring somatic mutations that were present in at least two samples. For each gene of interest, proteomic samples were categorized into mutated and wild‐type groups, and the fold change in protein expression was calculated. Differentially enriched proteins with a *P*‐value < 0.05 were further classified into two categories: those showing cis‐effects (directly influenced by mutations in the same gene) and those showing trans effects (influenced by mutations in other genes). Correlations between copy number alterations (CNA) and proteome were determined using Spearman correlation of common genes present in CNA‐proteome (6675 genes). In addition, *P*‐values (corrected for multiple testing using Benjamini–Hochberg FDR) for assessing the statistical significance of the correlation values were also calculated. CNA trans effects for a given gene were determined by identifying genes with statistically significant (*P*‐value < 0.05) positive or negative correlations.

## 3 Results

### 3.1 Somatic mutation landscape of cervical cancer

**Fig. 1:**
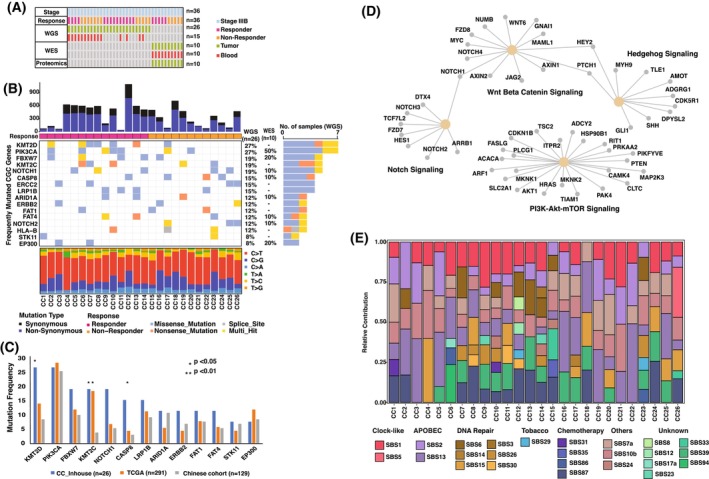
Somatic mutation landscape of cervical cancer in the Indian population. (A) Summary of the study dataset by cancer stage, treatment response, and genomic/proteomic analyses. (B) Mutation landscape of cervical cancer samples, highlighting frequently mutated cancer‐related genes. The top panel shows mutations per sample, followed by treatment response, and the bottom panel shows mutation type distribution. The right bar chart represents gene mutation frequencies across the cohort. (C) Comparison of mutation frequencies of recurrently mutated CGC genes between the Indian cohort and publicly available TCGA and Chinese cohorts. Statistical comparisons were performed for each gene individually between the Indian cohort and each of the other cohorts [*P < 0.05; **P < 0.01 (Fisher's Exact Test)]. (D) Enriched oncogenic signaling pathways based on the mutated genes and the genes involved in each pathway. (E) Relative contribution of COSMIC SBS signatures in the study cohort. Each bar represents an individual sample, with segments showing the proportion of COSMIC signatures categorized by etiological factors. CGC, cancer gene census; SBS, single base substitutions.

Twenty‐six cervical cancer stage IIIB samples, comprising 14 chemoradiation‐sensitive and 12 chemoradiation‐resistant cases, were subjected to WGS, while an additional 10 samples (five chemoradiation‐sensitive and five chemoradiation‐resistant) were subjected to WES (Fig. 1A). For WGS, the mean sequencing depth was 29.34× for tumors (ranging from 18.5× to 46.1×) and 31.63× for matched blood samples (ranging from 19× to 43.3×) (Table S2). WGS data analysis identified a total of 9917 single nucleotide variants (SNVs), including 6998 nonsynonymous variants and 2919 synonymous variants, with a median of 381 mutations per sample (ranging from 41 to 1093 mutations). The average TMB across the WGS cohort was 6.5 Muts/Mb (range: 0.64–17.07 muts/Mb). In comparison, WES data analysis identified a median of 160 mutations per sample, with a lower TMB of 4 mutations per Mb (Fig. S3A). By cross referencing WES data with WGS, we ensure to capture the critical variants facilitating wider interpretation and functional prediction.

The most prevalent somatic pathogenic genomic alterations were observed in *KMT2D* (27%) and *PIK3CA* (27%) followed by *FBXW7* (19%), *KMT2C* (19%), *NOTCH1* (19%), and *CASP8* (15%) (Fig. 1B). In addition, the analysis identified frequent mutations in *PLEC*, *EPPK1*, *MACF1*, *PKHD1L1*, and *DNAH11* genes, which are not reported to be significantly mutated genes associated with cervical carcinogenesis (Fig. S4, Table S3). A comparison of genomic alteration frequencies of the top mutated genes between our cohort and TCGA cohort (*n* = 291) and Chinese cohort (*n* = 129) presented in Fig. 1C revealed that *KMT2D*, *KMT2C*, and *CASP8* mutations were significantly more frequent in the Indian population than in the other two groups. In the WES analysis, *PIK3CA* was the most frequently mutated gene, identified in 50% of the samples. Among the 100 mutated genes from the CGC, 15 were mutated in more than two samples, including crucial cancer‐related genes, such as *ERBB4*, *KRAS*, *EP300*, *FBXW7*, *NCOR1*, *POLE*, and *ATR* (Fig. S3B). These genes are involved in key oncogenic pathways, including PI3K‐RTK‐RAS signaling, Notch signaling, and DNA repair (Fig. S5). To identify potential cancer driver genes in our cohort, we used the dNdScv model that quantifies the ratio of nonsynonymous to synonymous SNVs. *PIK3CA* and *HLA‐B* genes were statistically significant with *q* < 0.1. Nine missense mutations were identified across seven samples, with eight in the PI3Ka helical domain (E542K, E545K) and one in the PI3K_C2 domain (E453K) (Fig. S6). Mutational analysis showed significant enrichment in key oncogenic pathways, including PI3K‐AKT, NOTCH, Hippo, WNT, MAPK, and RAS signaling (Fig. 1D).

A comprehensive analysis of somatic mutation patterns in our cohort revealed a predominance of C>T transitions (55.3%) followed by C>G transversions (22%) (Fig. 1B, Bottom panel). To gain insight into the underlying mutational processes, we analyzed the trinucleotide context surrounding each substitution, and the resulting two mutational signatures (Fig. S7) were compared with known COSMIC signatures. APOBEC‐related signatures SBS2 and SBS13, characterized by C>G and C>T substitutions, were present in 84% and 92% of patients, reflecting APOBEC cytidine deaminase activity. Additionally, a clock‐like aging signature (SBS1) associated with spontaneous deamination of 5‐methylcytosine was detected in 88% of patients. Interestingly, SBS7a, typically linked to UV light exposure and predominantly found in melanoma, was detected in several samples in our cohort. This finding contrasts with previous reports in cervical cancer, and since cervical tissue is not directly exposed to UV light, the underlying cause of this signature remains unclear and warrants further investigation. However, oxidative stress, which is generally induced by persistent HPV infection, has been reported to generate DNA damage resembling UV‐like mutational patterns [33, 34, 35], which may plausibly contribute to the observed SBS7a signature. This warrants further investigation. Furthermore, signatures associated with defective DNA repair, SBS6 and SBS15, were identified in 42% of patients, emphasizing the critical role of DNA repair pathways in the genomic instability of cervical cancer. In addition, the SBS29 signature, linked to tobacco chewing, was detected in three patients (Fig. 1E).

### 3.2 Landscape of copy number alterations and structural variants in cervical cancer

**Fig. 2:**
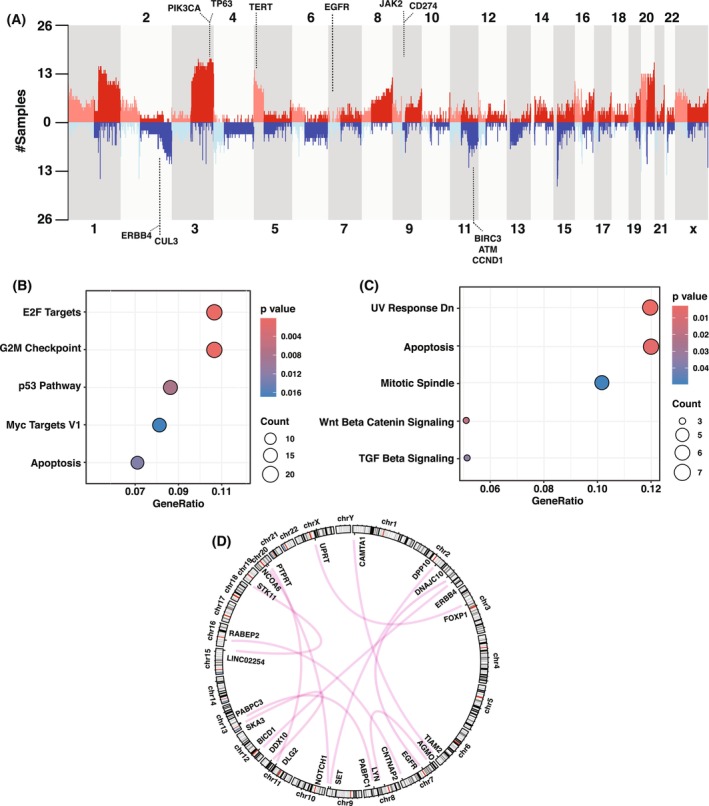
Identification of copy number alterations (CNAs) and structural variants. (A) Frequency of CNAs across chromosomes in cervical cancer samples. The *y*‐axis indicates the frequency of samples exhibiting copy number gains (red) or losses (blue) at each chromosomal location. Bubble plots illustrating the top five enriched hallmark pathways from MsigDB pathway analysis for genes amplified (B) and deleted (C) in cervical cancer samples. (D) Circos plot depicting interchromosomal translocations among CGC genes in our study cohort. CGC, cancer gene census.

Copy number analysis of WGS data using CNVKit identified a median of 130 amplifications per sample (range: 58–671), and a median of 101 deletions per sample (range: 65–234) (Table S4). Furthermore, the GISTIC2.0 algorithm was applied to identify somatic CNAs that might be responsible for driving tumorigenesis, revealing 89 focal events, comprising 25 amplification peaks and 54 deletion peaks (*q* < 0.25) (Fig. S8). Frequent gains occurred on chromosomes 3p, 7p, 11p, 19q, and 20q, and losses on 2q, 5q, 11q, 13q, and 17p, encompassing key oncogenes and tumor suppressor genes, such as *PIK3CA*, *TP63*, *EGFR*, *ATR*, *POLE*, *ERBB4*, *AKT2*, *ATM*, and *CCND1* (Fig. 2A). In the WES analysis, recurrent amplifications were similarly observed on chromosomes 3q, 19q, and 20q, while deletions were noted on chromosomes 2q, 11q, and 13q. These overlapping recurrent alterations highlight the critical role of these genomic regions in the progression of cervical cancer. The amplified cancer‐associated genes were enriched in pathways related to cell cycle regulation, tumor suppression, and DNA damage response, and apoptosis (Fig. 2B). Conversely, the deleted genes were predominantly associated with disruptions in pathways, including UV response downregulation, apoptosis, mitotic spindle integrity, and key signaling pathways, such as Wnt/β‐catenin and TGF‐β signaling (Fig. 2C). Utilizing the CNA classification framework, we identified two distinct CNA signatures within our cohort. A comparative analysis with the Pan‐Cancer Analysis of Whole Genomes (PCAWG) CNA signatures revealed significant similarity to CNS7 and CNS14 (cosine similarity > 0.5), which are characterized by small DNA segment alterations with minor copy number changes (Fig. S9). We identified a total of 3037 somatic SVs in our cohort, comprising 1615 deletions, 436 duplications, 427 inversions, and 559 translocations, with a median of 80 events per sample (range: 30–1800) (Table S5). Notably, among the 559 translocations, at least one breakpoint was located within 385 exonic regions, including several cancer‐associated genes, such as *ERBB4*, *STK11*, *NOTCH1*, *EGFR*, and *PTPRD* (Fig. 2D). The most frequently affected gene was TTC28 with eight unique rearrangements observed in six patients, likely reflecting underlying genomic instability. Prior studies suggest that such rearrangements are likely a consequence of genomic instability, potentially driven by LINE‐1 retrotransposon activity [36, 37].

### 3.3 Genomic alterations in chemoradiation‐resistant cervical cancer patients

**Fig. 3:**
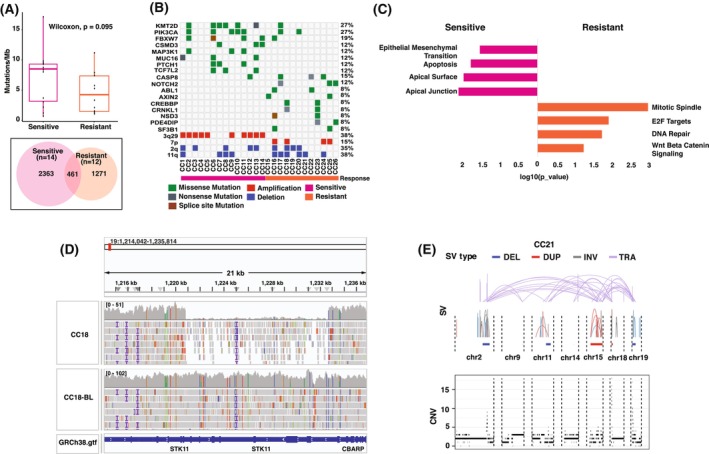
Genomic alterations in chemoradiation‐sensitive and chemoradiation‐resistant cervical cancer cohorts. (A) (Top) Box plot comparing TMB between two cohorts. (Bottom) Venn diagram showing shared and unique mutated gene counts between sensitive and resistant cohorts. (B) Heatmap showing the SNVs and CNVs recurrently altered in sensitive and resistant cohorts. (C) Bar graph showing the significantly enriched pathways based on the genes that are uniquely mutated in sensitive and resistant cohorts. (D) Integrative genome viewer snapshot illustrating the STK11 structural variant deletion detected in the chemoradiation‐resistant sample (CC18), contrasted with the paired blood sample showing no deletion. (E) SV and CN profiles of complex genomic rearrangements in CC21, showing copy number oscillations across three levels. Interchromosomal SVs (colored dots) and intrachromosomal SVs (black dots) are mapped onto the SV profile, with SV types represented by colored arcs. Seed regions (red bars) and linked regions (blue bars) are highlighted. CNVs, copy number variations; DEL, deletion; DUP, duplication; INV, inversion; SNVs, single nucleotide variants; SV, structural variants; TMB, tumor mutation burden; TRA, translocation.

To elucidate the genomic alterations associated with response to CCRT, we compared the mutational landscapes between chemoradiation‐sensitive and chemoradiation‐resistant patients. Comparison of TMB between the groups showed elevated median mutational burden (8.5 muts/Mb) in the sensitive group compared with the resistant group (4.25 muts/Mb); however, the difference was not statistically significant (*P* = 0.09, Wilcoxon test) (Fig. 3A). Notably, the sensitive and resistant groups showed distinct mutational profiles. In the sensitive group, *KMT2D* was the most frequently mutated gene, observed in 43% of patients, followed by PIK3CA (36%), *FBXW7* (29%), and *MAP3K1, PTCH1*, and *TCF7L2* (21%). In contrast, the resistant group displayed fewer recurrently mutated driver genes, with *NOTCH2* and *CASP8* mutated in 21% of patients (Fig. 3B). Pair‐wise mutual exclusivity and co‐occurrence analysis of the mutated genes across both cohorts revealed significant co‐occurrence of the *SYNE2‐PIK3CA* and *SYNE2‐FAT2* gene pairs (*P* < 0.05) in the sensitive cohort and *NOTCH2‐EPPK1* and *HLA‐B‐MACF1* (*P* < 0.05) pairs in the resistant cohort (Fig. S10).

To explore differences in copy number patterns between the sensitive and resistant cohorts, we conducted GISTIC analysis separately for each cohort (Fig. S11). The sensitive group had nine recurrent focal amplifications, while the resistant group showed only one peak on 7p, encompassing *EGFR*, a known driver of tumor proliferation. We further extended our analysis to examine TCGA cervical cancer data for *EGFR* RNA expression and observed significantly higher expression in the *EGFR*‐amplified group (Fig. S12A). EGFR amplification was also associated with shorter PFIs, suggesting its role in chemoradiation resistance and poorer outcomes (Fig. S12B). Pathway enrichment analysis on the uniquely mutated and amplified genes revealed significant overrepresentation of DNA repair and Wnt/β‐catenin signaling pathways in the resistant cohort, underscoring their roles in genomic stability and cellular proliferation (Fig. 3C and Table S6). Structural variants investigation did not identify any recurrent fusions in either cohort. However, *STK11*, a key tumor suppressor gene, was uniquely deleted in the resistant group (Fig. 3D). TCGA cervical cancer data confirmed significantly reduced RNA expression and poorer disease‐free survival in the *STK11* deletion group (Fig. S12C,D). We further investigated chromothripsis events in both cohorts based on CNAs and structural variants. Complex genomic rearrangements in three of the 12 resistant patients (Fig. 3E and Fig. S13A,B). CGR signature classification revealed distinct patterns in each patient, including chromothripsis driven by chromatin bridge breakage and BFB cycles (Signature 2), micronuclei formation (Signature 4), and uneven breakpoints with minimal CNAs (Signature 6) (Fig. S13C). These findings underlined the potential role of chromothripsis in driving genomic instability and contributing to chemoradiotherapy resistance in a subset of cervical cancer patients.

### 3.4 Proteomic landscape of chemoradiation‐resistant cervical cancer patients

**Fig. 4:**
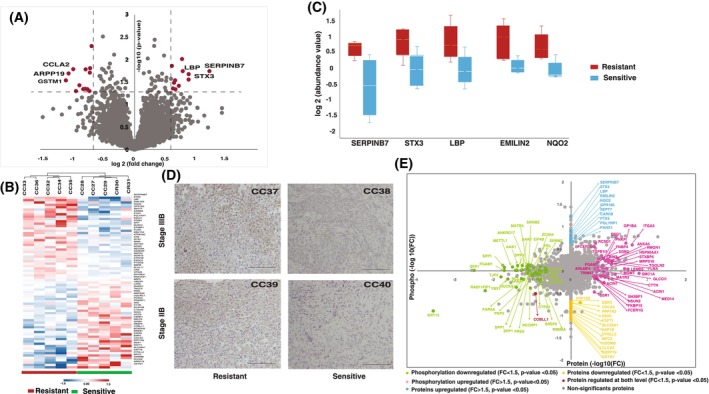
Global proteome of chemoradiotherapy resistant cervical cancer patients. (A) Volcano plot of quantified proteins in the resistant and sensitive cohorts. Dashed lines represent significance levels. (B) Heatmap illustrating the significantly expressed proteins in the resistant cohort compared with the sensitive cohort. (C) Box plot representing the top five upregulated proteins in the resistant cohort compared with the sensitive cohort based on their log2 abundance values. Horizontal lines within each box indicate the median values. Error bars represent standard deviation (SD). (D) Immunohistochemical analysis showing STX3 expression in cervical cancer tissues from nonresponders and responders. Scale bar represents 100 μm. (E) Scatter plot comparing the protein expression and phosphorylation. Phosphorylation fold change (−log10) plotted against protein fold change (−log10).

Following the genomic analysis, we further carried out proteomic profiling of treatment‐resistant and sensitive patients to understand the proteomic signatures associated with treatment resistance. To identify the altered protein expression associated with CCRT resistance, we carried out multiplexed TMT and LC–MS/MS‐based global proteomics of treatment‐naïve cervical cancer patients. The cervical cancer samples (five chemoradiation‐sensitive and five chemoradiation‐resistant), which were subjected to WES, were also profiled for proteome analysis, leading to the identification of 8373 proteins and the quantification of 7138 proteins across all samples (Fig. 4A and Table S7). The statistical analysis of the two cohorts revealed significant expression of 73 proteins with a *P*‐value cutoff of 0.05. The hierarchical clustering of the significantly expressed proteins revealed a distinct proteomic signature between the two cohorts (Fig. 4B). We observed dysregulation of 24 proteins with a fold change cutoff of 1.5, of which 11 proteins showed high expression and 13 proteins showed low expression, respectively, in the resistant cohort. The proteins such as SERPINB7, STX3, LBP, EMILIN2, and NQO2 were the top five highly expressed proteins in chemoradiation‐resistant patients (Fig. 4C). We further checked the mRNA expression of the dysregulated proteins in the cervical cancer patients showing differential expression between the tumor and normal subjects (Fig. S14).

Furthermore, we extended our study to validate the differential expression of syntaxin 3 (STX3), a member of the syntaxin family and is involved in exocytosis as well. Based on our data, we sought to determine the differential expression of STX3 in a larger cohort using immunohistochemistry. We collected FFPE tissue samples of resistant and sensitive cohorts of stage IIB as well as stage IIIB disease. For the validation experiment, we included 16 patients (eight sensitive and eight resistant samples) of stage IIB and 16 patients from stage IIIB (eight sensitive and eight resistant samples). In concordance with the proteomic data, we observed high expression of STX3 in stage IIIB resistant patients. Furthermore, we extended our cohort selection to stage IIB patients also, and we observed the concordant finding in the expression (Fig. 4D). The pathway analysis of significantly expressed proteins revealed the enrichment of pathways such as the establishment of sister chromatid cohesion, MASTL facilitates mitotic progression, biological oxidation, resolution of sister chromatid cohesion, and regulation of TLR by endogenous ligand (Fig. S15). We integrated our recently published phosphoproteomics data for the same set of samples with the existing proteomic dataset and 1365 proteins were commonly identified between the two data sets [38]. Seventy‐two and one hundred twenty‐two proteins were significantly regulated at the level of protein expression and phosphorylation, respectively (Fig. 4E).

### 3.5 Integrated proteogenomic analysis

**Fig. 5:**
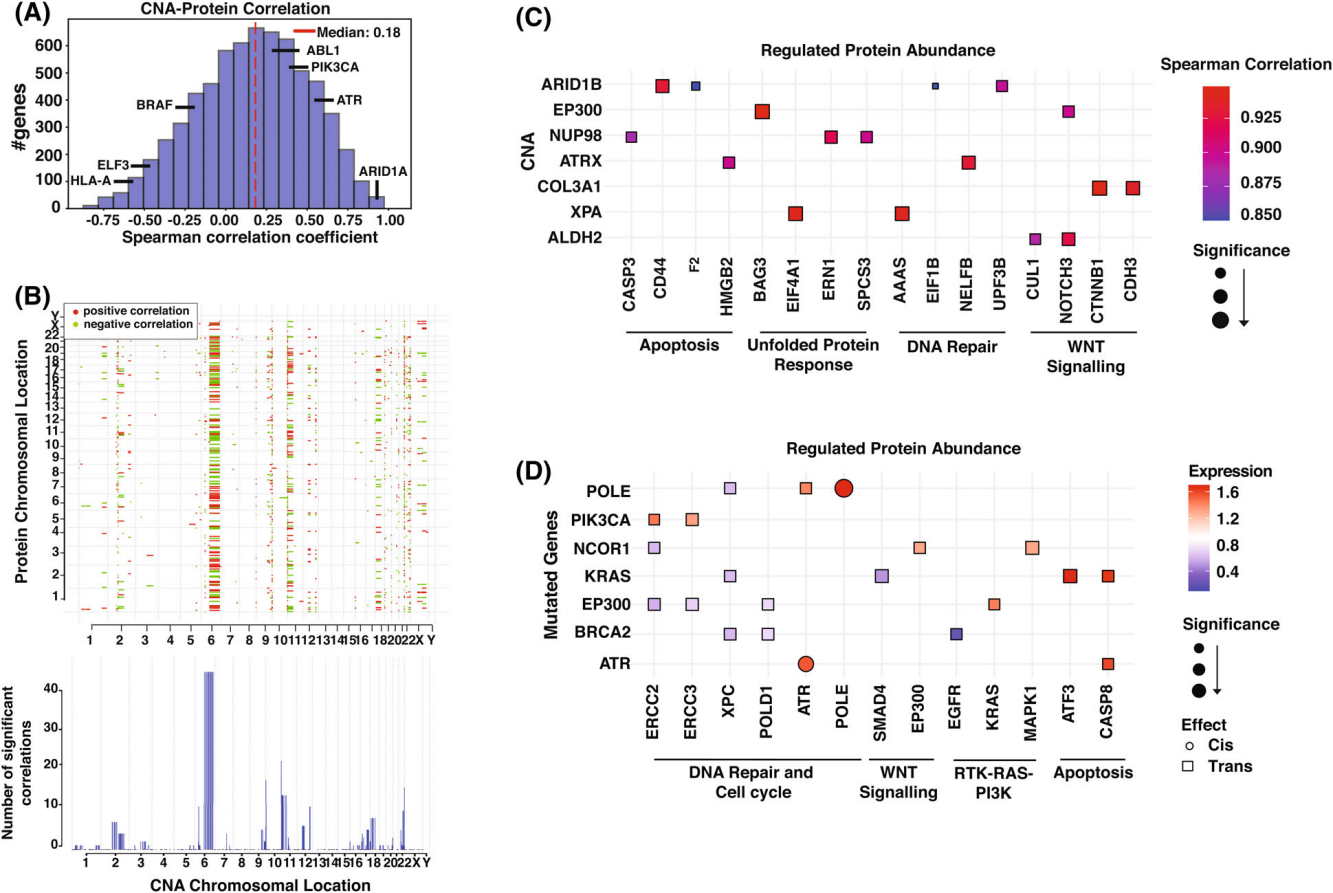
Impact of genomic alterations in protein expression. (A) Histogram showing the distribution of per‐gene Spearman correlation coefficients between copy number alterations and protein expression. (B) Effects of CNA on protein levels. Top: copy number correlation with protein. Bottom: the frequency of copy number correlation with protein. (C) Trans effect of CNA on protein expression. (D) Cis and trans effects of genomic mutations on protein levels. CNA, copy number alteration.

To understand the downstream effects of genomic alterations on cellular pathways and protein interaction, we integrated whole exome and proteomic data to analyze cis and trans effects of genetic variants. This comprehensive approach enables us to investigate how genetic variations directly (cis) or indirectly (trans) influence protein expression and function. Correlation analysis between paired CNA and proteomics datasets revealed that 66.96% of the 6775 CNA–protein pairs were positively correlated in tumor samples, though no significant cis‐regulatory effects were observed (FDR < 0.1) (Fig. 5A). Notably, Fig. 5B highlights several trans‐acting CNA hotspots, which are chromosomal regions where alterations significantly affect the expression of proteins at distant genomic loci. Chromosomes 6q, 9q, 11p, and 22q showed significant trans‐correlations, with the 6q region alone influencing the expression of over 200 proteins. Further investigation of cancer‐associated genes within these CNA regions identified 107 genes that were positively correlated with protein expression via trans effects, impacting pathways, such as apoptosis, unfolded protein response, DNA repair, WNT/β‐catenin signaling, and Notch signaling (Fig. 5C).

We extended our investigation into the impact of gene mutations on protein expression, focusing on cancer‐related genes that were mutated in two or more samples. Significant changes in protein expression were observed, either within the same gene (cis‐effects) or in other genes (trans effects) (*P* ≤ 0.05). Most of the mutated genes influenced the expression of one or more proteins involved in the DNA repair pathway. Additionally, key pathways, such as cell cycle regulation (*ATR*), PI3K‐RTK‐RAS signaling (*KRAS*, *MAPK1*), and apoptosis (*CASP8*), were affected mainly through trans effects, where mutations in one gene influenced the expression of other genes within these pathways. Notably, cis effects were observed in *ATR* and *POLE*, where mutations directly influenced their own protein expression (Fig. 5D). These findings suggest that these genomic alterations are driving tumor progression through both direct and indirect mechanisms, affecting crucial oncogenic signaling pathways, including PI3K‐RTK‐RAS, cell cycle, DNA repair, and apoptosis.

### 3.6 Clinically actionable mutation in cervical cancer

Actionable targets from the cervical cancer samples (WGS/WES, *n* = 36) were identified using the OncoKB database, which classifies evidence for actionability into seven levels. In this cohort, actionable mutations were found across four OncoKB evidence levels (1, 2, 3A, and 4) [32]. Specifically, 33% of the samples harbored Level 1 actionable evidence, 11% exhibited Level 2, 25% had mutations classified as Level 3A, and 5% were categorized under Level 4 (Fig. S16A). We observed *EGFR* amplification in six chemoradiation‐resistant patients, indicating a potential actionable target. According to Level 3 evidence, *EGFR* amplification is successfully treated with cetuximab or panitumumab in combination with chemotherapy in esophagogastric cancer, suggesting an alternate therapeutic approach for CCRT in chemoradiation‐resistant cervical cancer patients. Additionally, *PIK3CA*, frequently mutated in cervical cancer, is currently targeted in breast cancer with FDA‐approved drugs Alpelisib + Fulvestrant and Capivasertib + Fulvestrant (Fig. S16B). Given its prevalence, *PIK3CA*‐targeted therapies represent another promising avenue for personalized treatment in cervical cancer.

## 4 Discussion

We present the first comprehensive WGS‐based analysis of somatic genome alterations in Indian cervical cancer patients, uniquely demonstrating a broad and in‐depth analysis of treatment response‐related alterations in chemoradiation‐resistant and sensitive cohorts. Additionally, we conducted proteomic profiling on a subset of samples from both resistant and sensitive groups, providing an integrated view of the genomic and proteomic landscape associated with chemoradiation response in this population. Our analysis revealed recurrent mutations in key genes, such as *PIK3CA*, *KMT2D*, *FBXW7*, *KMT2C*, and *NOTCH1*. The mutation spectrum was largely comparable to previous studies involving diverse populations, highlighting shared oncogenic drivers in cervical cancer [13, 39]. *PIK3CA* emerged as the frequently mutated gene, which is consistent with previous studies in squamous cervical carcinoma and other solid tumors. Notably, *PIK3CA* mutations were predominantly concentrated in the helical domain (E542:E545), a hotspot that promotes PI3Kα activation, thus enhancing the PI3K/AKT signaling pathway, which is known to contribute to carcinogenesis, cellular growth, and proliferation [40]. Henderson et al. [41] further demonstrated that the high frequency of these helical domain hotspot mutations is driven by APOBEC‐mediated mutagenesis in HPV‐driven tumorigenesis. However, no significant difference was observed in the *PIK3CA* mutation frequency between chemoradiation‐resistant and sensitive cohorts. The impact of *PIK3CA* mutations on cervical cancer outcomes has been widely debated, with studies showing both negative and positive associations with survival [42, 43, 44, 45]. This complexity highlights the need for further investigation into the role of *PIK3CA* in cervical cancer progression and treatment response. Another frequent mutation observed in our cohort involves chromatin remodeling genes, particularly *KMT2D* and *KMT2C*, with *KMT2D* mutations being more common in the chemoradiation‐sensitive group. Loss of function in *KMT2A‐D* genes leads to epigenetic modifications and defective H3K4 methylation in cervical cancer [46]. Another genomic study reported the association of the *KMT2D* mutation with high TMB and improved response to immune checkpoint inhibitor therapy in cervical cancer [47]. The higher *KMT2D* mutation frequency in the Indian population suggests its role in shaping the cervical cancer epigenetic landscape and influencing treatment response.

The enrichment of amplified genes highlights the dysregulation of key pathways, including cell cycle control, DNA repair, and apoptosis, promoting cell proliferation, genomic instability, and contributing to the pathogenesis of cervical cancer [48]. *EGFR* expression in the 7p region is more prominent in squamous cell carcinoma compared with other histological subtypes [49]. In this study, 15% of patients exhibited *EGFR* amplification in the resistant cohort, indicating that *EGFR* may be a critical therapeutic target for this group. A previous study has also shown that high *EGFR* expression is associated with poor disease‐free survival (DFS) in squamous cell carcinoma of cervical tumors [50]. *EGFR* blockade has been shown to increase radiosensitivity *in vitro* by modulating apoptosis and proliferation, and it showed increased radio‐response *in vivo* [51, 52]. While cetuximab, an EGFR‐targeting monoclonal antibody, has demonstrated efficacy in combination with chemoradiation for head and neck cancer, its impact on cervical cancer remains unexplored [53]. A randomized phase II trial showed no significant improvement in survival outcomes when cetuximab was added to standard chemotherapy for advanced or recurrent cervical cancer; however, it was ambiguous as the patient selection criteria were not based on *EGFR* amplification [54]. In contrast, a phase I/II trial combining erlotinib with cisplatin‐based chemoradiotherapy for LACC demonstrated a high response rate (94.4%) and long‐term survival, suggesting that EGFR inhibition may enhance chemoradiation efficacy [55]. Notably, in a recent case report, afatinib, a second‐generation tyrosine kinase inhibitor, demonstrated efficacy as a fourth‐line treatment for an *EGFR*‐amplified metastatic cervical cancer patient, resulting in a partial response and a progression‐free survival of 5.5 months [56]. Another interesting finding from our study is the structural variant deletion of the tumor suppressor gene *STK11* in four chemoradiation‐resistant patients, underscoring its potential role in mediating treatment resistance. Consistent with our findings, previous studies have correlated *STK11* alterations, including copy number loss or mutation, with reduced overall survival and poor prognosis [39, 57]. The loss of *STK11* function may contribute to radio‐resistance by upregulating antioxidant enzymes via NRF2, thereby mitigating ROS‐induced damage. Preclinical and clinical evidence indicates that *STK11*‐deficient tumors exhibit poor responses to radiotherapy and are associated with shorter disease‐free survival.

Mass spectrometry‐based proteomics led to the identification of key proteins associated with chemoradiation resistance in cervical cancer, confirming the potential of proteomic profiling to differentiate patients based on treatment response. The functional analysis of the proteins revealed the establishment of sister chromatid cohesion and is regarded for its involvement in chromosome segregation and its role in homologous recombination of DNA double strand break repair [58]. Among the dysregulated proteins, we identified SERPINB7 and STX3, previously linked to poor outcomes in various cancers, including cervical cancer [59, 60]. Immunohistochemistry confirmed the elevation of STX3 in the resistant cohort, reinforcing its potential as a predictive biomarker for CCRT resistance. STX3, a SNARE family protein, regulates vesicle fusion and integrin trafficking, and its knockdown has been shown to reduce cancer cell migration and survival [61]. High STX3 expression also correlates with the activation of the PI3K‐AKT‐mTOR pathway, frequently dysregulated in cervical cancer and associated with treatment resistance [62]. Additionally, our validation in stage IIB patients showed a similar trend, further substantiating the utility of STX3 as a predictive biomarker.

**Fig. 6:**
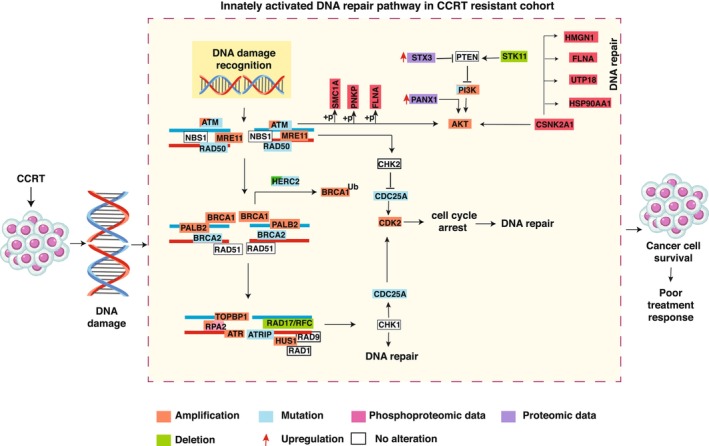
Data‐driven pathway illustrating the innately activated DNA repair mechanism in chemoradiation‐resistant cervical cancer. The figure illustrates the molecular alterations observed in the DNA damage recognition and repair pathways in patients' resistant to concurrent chemoradiation therapy (CCRT). DNA damage induced by CCRT activates the ATM/ATR signaling cascade, leading to the recruitment and activation of multiple repair proteins, such as BRCA1/2, RAD51, and CHK1/2. In the resistant cohort, multiple components of the DNA repair pathway exhibit genomic (amplifications, mutations, and deletions) and proteomic (expression and phosphorylation) alterations. Notably, genes, such as CSNK2A1, PI3K, and AKT, are implicated in enhancing DNA repair and promoting cell survival, contributing to treatment resistance. Upregulation of STX3 and loss of tumor suppressors, such as PTEN, further enhance PI3K/AKT signaling. Proteins with amplification (orange), mutation (blue), deletion (green), phosphoproteomic (pink), and proteomic (purple) alterations are marked. Arrows denote upregulation, while ubiquitination and phosphorylation events are also illustrated. This hyperactivation of the DNA repair pathway enables cancer cells to survive therapy‐induced damage, leading to poor treatment outcomes.

Pathway enrichment analysis revealed distinct biological processes associated with treatment response. In the sensitive cohort, apoptosis and epithelial–mesenchymal transition pathways were enriched. Although EMT has often been associated with poor prognosis in previous studies [13, 63], its enrichment in the sensitive cohort suggests a potentially distinct biological role that warrants further investigation. Pathway analysis from both genomic and proteomic data consistently highlighted the enrichment of the DNA repair pathway in the chemoradiation‐resistant cohort, underscoring its critical role in driving treatment resistance. Notably, these findings align with our recently published phosphoproteomics data where we have demonstrated the role of the DNA repair pathway in the resistant cohort. To gain a deeper insight on treatment resistance, we curated a data‐driven DNA repair pathway integrating genomic, proteomic, and phosphoproteomic data to assess their impact on cell signaling and disease progression in the resistant cohort (Fig. 6). This integrative analysis highlights an innately active DNA repair pathway in the resistant cohort, characterized by alterations in key components, such as *ATM*, *ATR*, *BRCA2*, and *RAD50*. Phosphoproteomic data revealed the activation of key proteins, such as CSNK2A1 and SMC1A, further supporting their roles in promoting tumor survival through efficient DNA repair [38]. Alterations in pathways, such as PI3K‐AKT signaling, driven by *STK11* deletion and elevated STX3 expression, might interact with DNA repair mechanisms, contributing to treatment resistance. Given their involvement in these interconnected pathways, *STK11* and STX3 emerge as promising predictive biomarkers for identifying patients at risk of poor therapeutic response to CCRT, guiding personalized treatment approaches. Amplifications, mutations, and dysregulations in DNA damage recognition and repair collectively facilitate enhanced DNA repair in resistant tumors, potentially enabling tumor cells to evade treatment‐induced DNA damage.

Our study is the first comprehensive genomic and proteomic profile of treatment resistant cervical cancer; however, it is constrained by sample size, which may limit the statistical power and applicability of the findings to broader patient populations. The short follow‐up period with low mortality in the patient cohort excluded the assessment of the relationship between these alterations and overall survival. Additionally, although this study identifies novel genomic alterations and candidate biomarkers, independent validation using larger cohorts and further functional studies will enhance clinical utility.

## 5 Conclusions

In conclusion, this comprehensive genomic and proteomic analysis provides critical insights into the molecular landscape of Indian cervical cancer patients, particularly in the context of chemoradiation response. Elevated STX3 expression is identified as a promising predictive biomarker for treatment resistance, while *STK11* deletion indicates poor chemoradiotherapy outcomes. Furthermore, *EGFR* amplification in the resistant cohort highlights its potential as a therapeutic target, offering new directions for precision medicine. These findings pave the way for personalized therapeutic strategies to enhance treatment efficacy, particularly in resource‐limited settings where the burden of cervical cancer is significant.

## Conflict of interest

The authors declare no conflict of interest.

## Author contributions

JS analyzed, compiled, and interpreted the data and wrote the manuscript. IAG generated and analyzed the proteomic data and contributed to the manuscript preparation. JS and IAG have contributed equally. SSM analyzed and interpreted the data and reviewed the manuscript drafts. PA generated the genomic data. ERD, KP, SL, RD, and DP provided the critical clinical feedback and reviewed the manuscript drafts. RSK acquired the data and provided the resources for the data analysis. MS and VT contributed to the patient sample acquisition and validated the samples. RC conceptualized the study, provided the clinical data, and reviewed the manuscript drafts. PK conceptualized the study, interpreted the data, and wrote and reviewed manuscript drafts. All authors reviewed and approved the manuscript prior to submission.

## Supporting information


**Fig. S1.** Workflow for TMT‐based quantitative proteomic analysis of cervical cancer tissues.
**Fig. S2.** Depth of coverage (DP) distribution for variants identified through WGS and WES.
**Fig. S3.** Somatic mutation burden and mutational landscape of cervical cancer whole exome sequencing (WES) cohort.
**Fig. S4.** Heatmap depicting the top 40 mutated genes in the whole genome sequencing cervical cancer cohort.
**Fig. S5.** Enrichment of Oncogenic Signaling Pathways in cervical cancer Whole Exome Sequencing (WES) cohort.
**Fig. S6.** Lollipop plot visualizing missense mutations identified in the cervical cancer cohort, annotated with cancer hotspots and OncoKB.
**Fig. S7.** Mutational signature analysis in cervical cancer.
**Fig. S8.** Copy number analysis in cervical cancer.
**Fig. S9.** Copy number signature analysis.
**Fig. S10.** Heatmap depicting the co‐occurring and mutually exclusive mutated genes across chemoradiation sensitive and resistant cohorts.
**Fig. S11.** Recurrent copy number alterations in chemoradiation sensitive and resistant cervical cancer cohorts.
**Fig. S12.** Expression and prognostic implications of EGFR amplification and STK11 structural variant deletion in cervical cancer cohort.
**Fig. S13.** Complex genomic rearrangements (CGRs) and signature analysis.
**Fig. S14.** The mRNA expression levels of the genes (SERPINB7, STX3, LBP, EMILIN2, and NQO2) in cervical squamous cell carcinoma (CESC) tissues compared to normal tissues.
**Fig. S15.** Functional enrichment analysis revealed the pathways involved in treatment resistance in cervical cancer patients.
**Fig. S16.** Clinically actionable variants and treatments.

**Table S1.** Summary of treatment response and analysis methods.
**Table S2.** Overview of sequencing quality and coverage metrics.
**Table S3.** List of somatic variants identified in cervical cancer samples following CADD‐based filtering.
**Table S4.** Copy number alterations identified in cervical cancer patients.
**Table S5.** Structural variants identified in cervical cancer patients.
**Table S6.** Pathways enriched in chemoradiation‐sensitive and ‐resistant cohorts based on genes uniquely altered in each group.
**Table S7.** Quantified proteins of concurrent chemoradiation‐resistant and sensitive stage IIIB cervical cancer patients.

## Data Availability

The proteomics data generated in this study are available via ProteomeXchange under the identifier PXD058817. Other datasets will be made available upon request to the corresponding author, in accordance with institutional and ethical guidelines. Summary‐level data supporting the findings are provided in the Supporting Information.
