# Supplementary material for: Integrated genomic and proteomic profiling reveals insights into chemoradiation resistance in cervical cancer
Source: Mol Oncol. 2026 Jan 26;20(3):709–26. doi: 10.1002/1878-0261.70108 (PMC13042866; doi:10.1002/1878-0261.70108)
Supplement: Supplementary file 1 — Fig. S1. Workflow for TMT‐based quantitative proteomic analysis of cervical cancer tissues. Fig. S2. Depth of coverage (DP) distribution for variants identified through WGS and WES. Fig. S3. Somatic mutation burden and mutational landscape of cervical cancer whole exome sequencing (WES) cohort. Fig. S4. Heatmap depicting the top 40 mutated genes in the whole genome sequencing cervical cancer cohort. Fig. S5. Enrichment of Oncogenic Signaling Pathways in cervical cancer Whole Exome Sequencing (WES) cohort. Fig. S6. Lollipop plot visualizing missense mutations identified in the cervical cancer cohort, annotated with cancer hotspots and OncoKB. Fig. S7. Mutational signature analysis in cervical cancer. Fig. S8. Copy number analysis in cervical cancer. Fig. S9. Copy number signature analysis. Fig. S10. Heatmap depicting the co‐occurring and mutually exclusive mutated genes across chemoradiation sensitive and resistant cohorts. Fig. S11. Recurrent copy number alterations in chemoradiation sensitive and resistant cervical cancer cohorts. Fig. S12. Expression and prognostic implications of EGFR amplification and STK11 structural variant deletion in cervical cancer cohort. Fig. S13. Complex genomic rearrangements (CGRs) and signature analysis. Fig. S14. The mRNA expression levels of the genes (SERPINB7, STX3, LBP, EMILIN2, and NQO2) in cervical squamous cell carcinoma (CESC) tissues compared to normal tissues. Fig. S15. Functional enrichment analysis revealed the pathways involved in treatment resistance in cervical cancer patients. Fig. S16. Clinically actionable variants and treatments. [file MOL2-20-709-s001.zip › Sambath et al_Supplementary Figures_FL.pdf]

Integrated genomic and proteomic profiling reveals insights into chemoradiation resistance in cervical cancer

Supplementary Figures

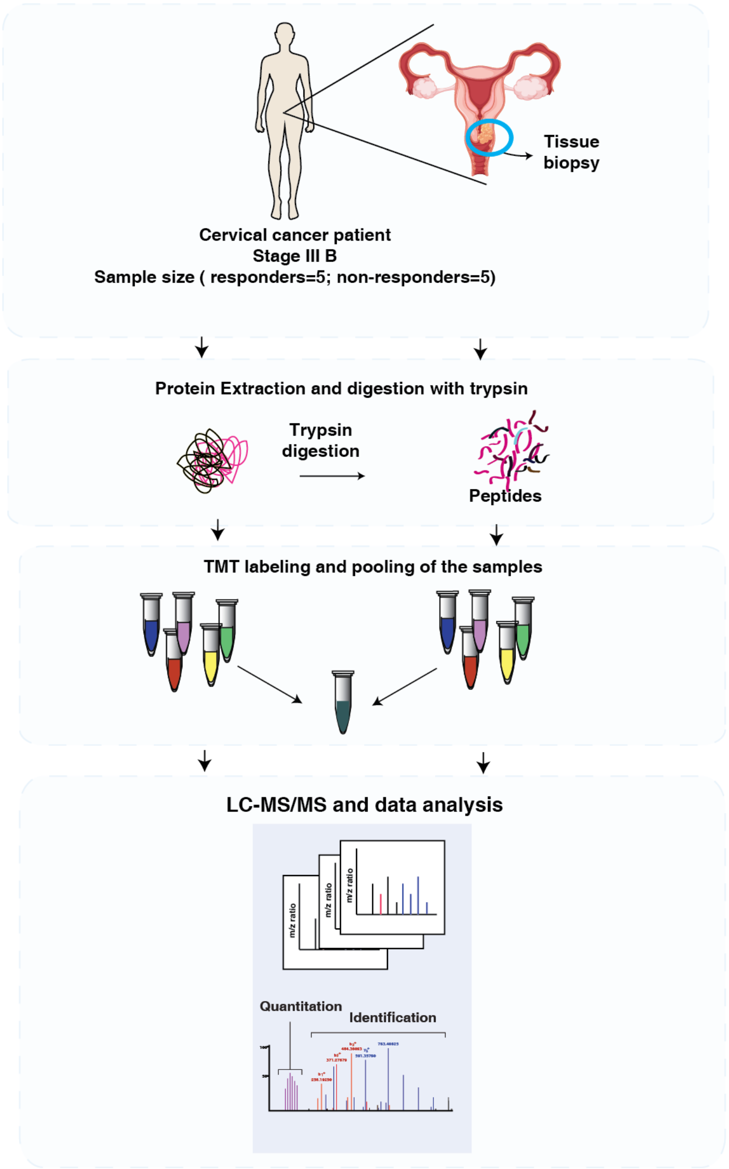

Supplementary Figure 1: Workflow for TMT-based quantitative proteomic analysis of cervical cancer tissues. Biopsy samples were obtained from Stage III B cervical cancer patients (n=10, including 5 responders and 5 non-responders). Protein extraction was performed, followed by trypsin digestion and the resulting peptides were labeled using 10-plex tandem mass tags (TMT) and pooled. The labeled peptides were analyzed using liquid chromatography-tandem mass spectrometry (LC-MS/MS), and the data were processed for protein identification and quantitation.

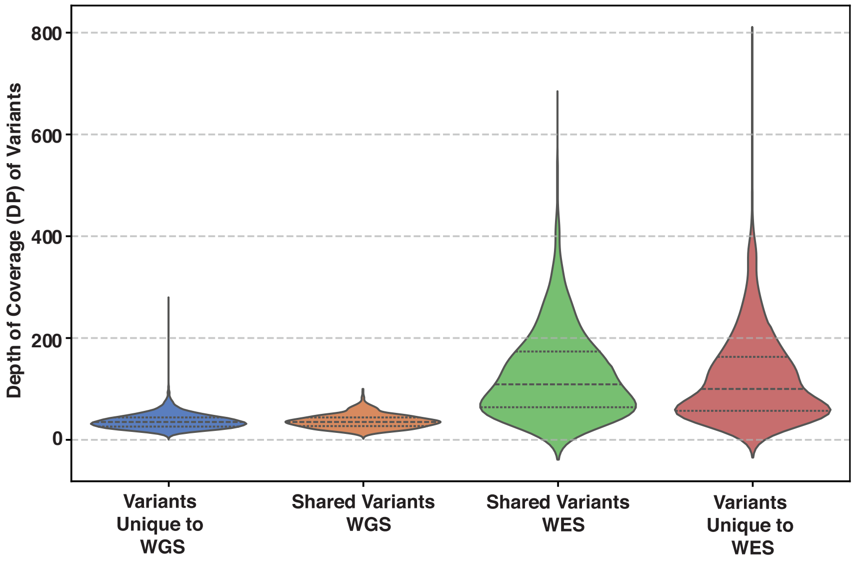

Supplementary Figure 2: Violin plot representing the depth of coverage (DP) distribution for variants identified through WGS and WES. The categories include variants unique to WGS, shared variants identified by both WGS and WES (represented separately for WGS and WES), and variants unique to WES. The y-axis depicts the depth of coverage (DP) of the variants, while the x-axis categorizes the variant groups. Each violin plot illustrates the distribution, median, and interquartile range of the DP.

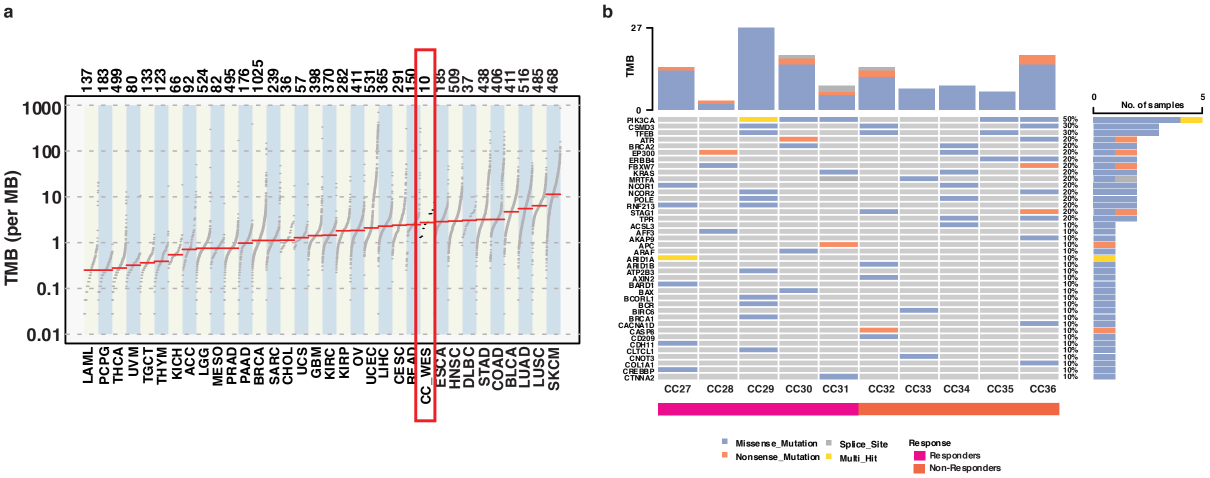

Supplementary Figure 3: Somatic Mutation Burden and Mutational Landscape of Cervical Cancer Whole Exome Sequencing (WES) Cohort**. (a)** The comparison of mutation burden of cervical cancer WES cohort across TCGA datasets. Our samples are indicated by red rectangle. Y-axis is the total number of non-synonymous mutations found in each tumor sample. **(b)** Heatmap demonstrating somatic mutation profiles of the top 40 genes identified from WES analysis. The left bar plot indicates the number of samples with mutations in each gene. The top panel shows the mutation burden per sample, while the bottom panel denotes the treatment response of each sample.

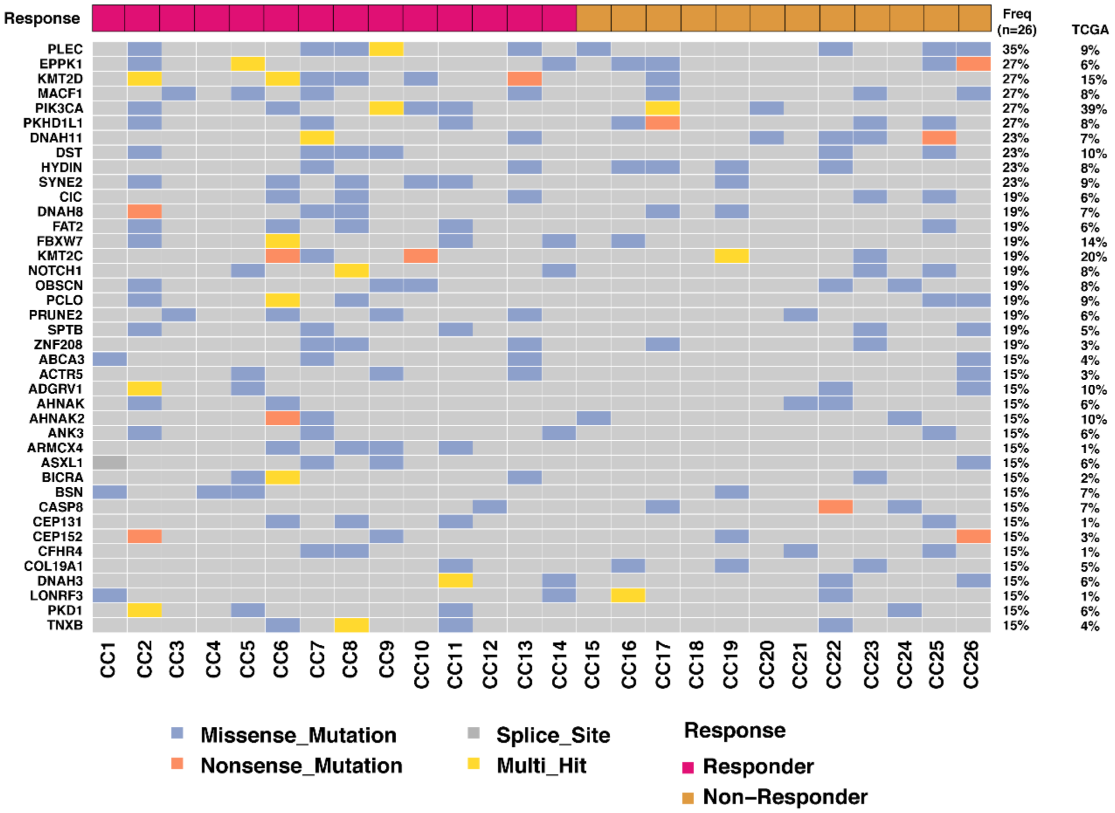

Supplementary Figure 4: Heatmap depicting the top 40 mutated genes in the WGS cervical cancer cohort.

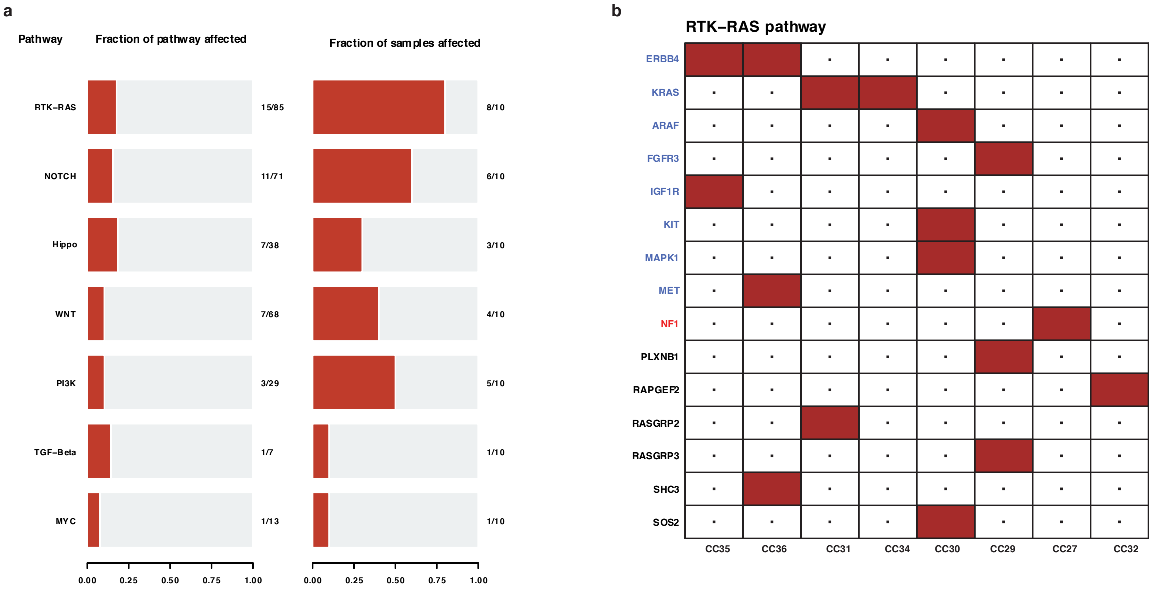

Supplementary Figure 5: Enrichment of Oncogenic Signaling Pathways in cervical cancer Whole Exome Sequencing (WES) cohort. **(a)** Enriched oncogenic signalling pathways in cervical cancer WES samples. The bar plots on the left show the number of mutated genes in

the pathway in each of the cohorts, and the bar plots on the right shows the fractions of samples having mutated genes in the pathway. **(b)** Oncoplot showing somatic mutations in genes involved in the RTK–RAS signaling pathway across in cervical cancer WES samples. Tumour suppressor genes are in red font and oncogenes are in blue font.

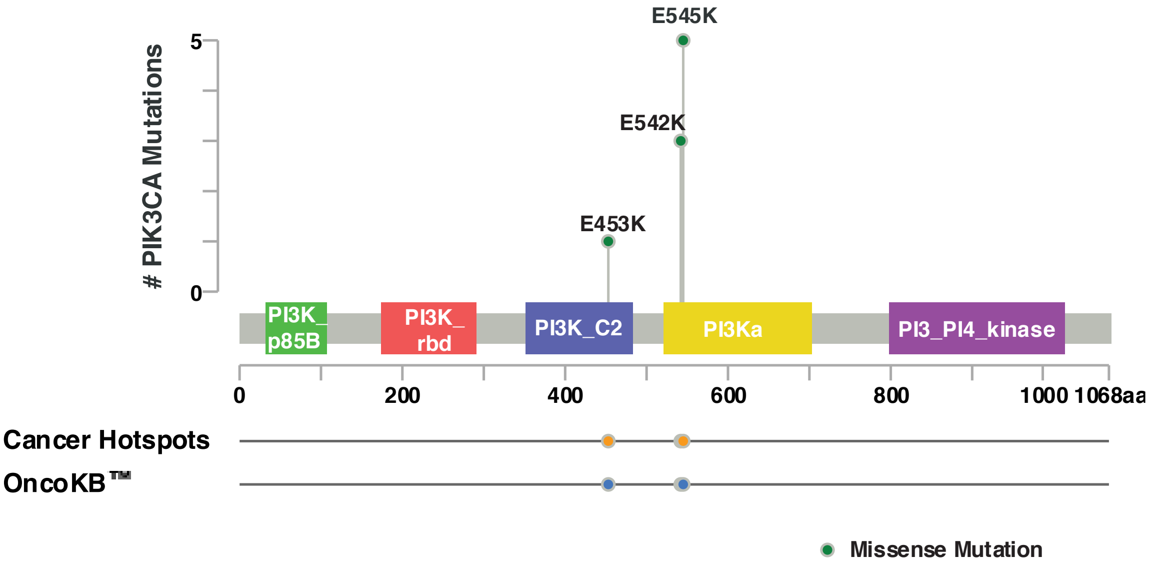

Supplementary Figure 6: Lollipop plot visualizing missense mutations identified in the cervical cancer cohort, annotated with cancer hotspots and OncoKB

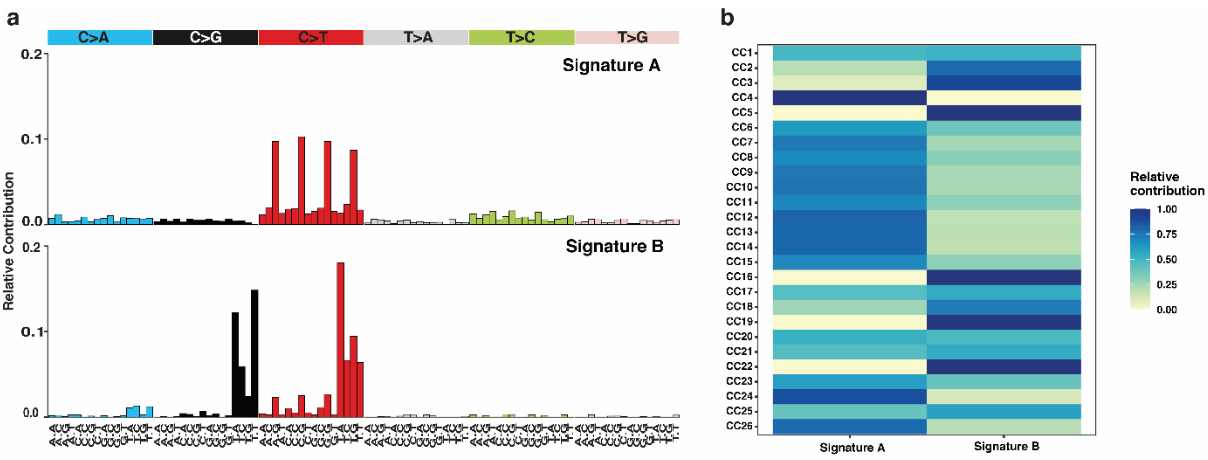

Supplementary Figure 7: Mutational signature analysis in cervical cancer. **(a)** Two mutation signatures generated from mutation types of all cervical cancer samples. Y-axis shows the relative contribution of the indicated mutation types in X axis from all the samples. **(b)** Relative contribution of derived mutational signatures in tumor samples; The y-axis shows the tumor sample id and the x-axis represents the mutational signature.

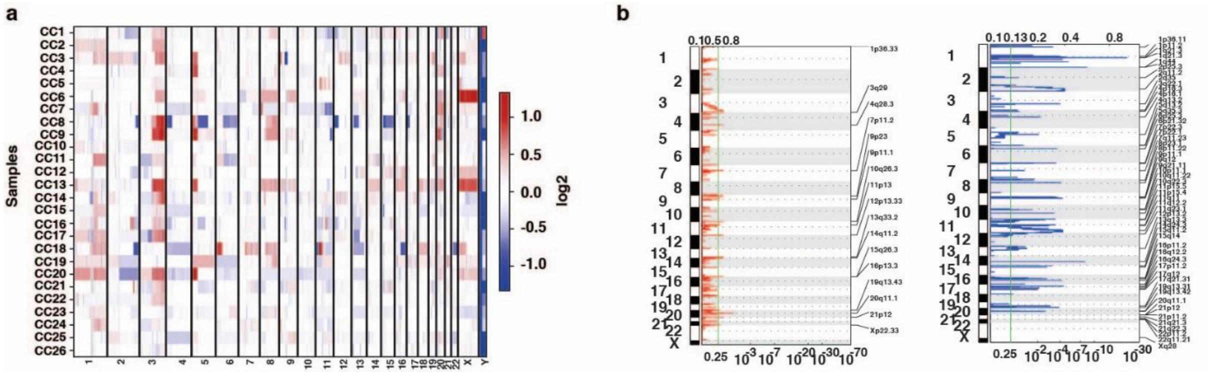

Supplementary Figure 8: Copy number analysis in cervical cancer. **(a)** Heatmap displaying the identified CNVs across cervical cancer cohort using CNVKit. **(b)** Recurrent copy number amplification and deletion identified by GISTIC 2.0. The genome is oriented vertically from top to bottom in the left and the green threshold line signifies the q-value threshold in the x-axis. Red peaks and blue peaks indicate amplifications and deletions, respectively.

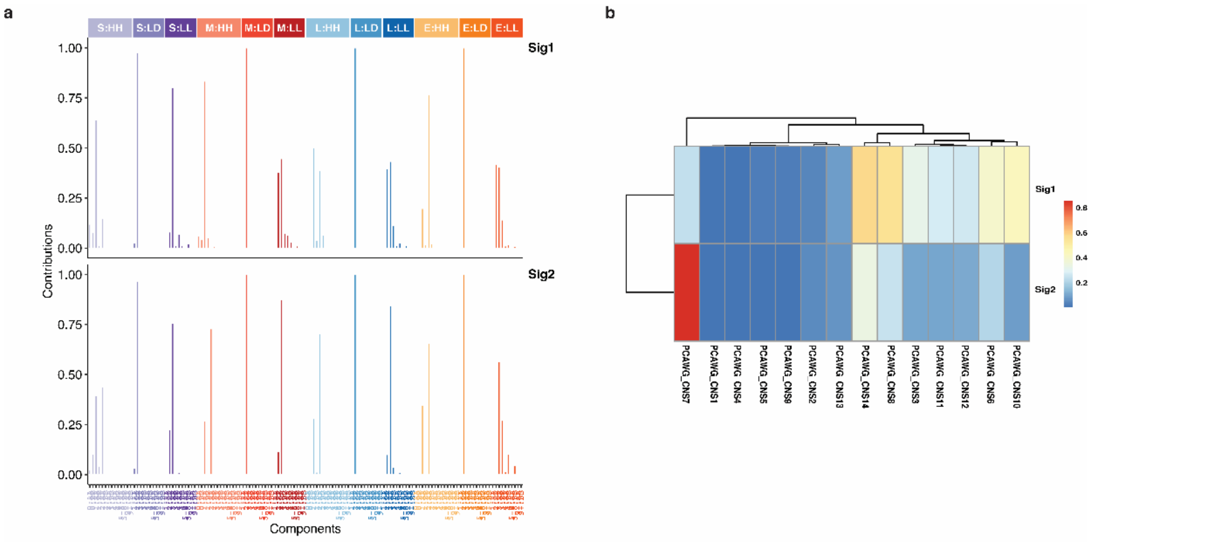

Supplementary Figure 9: Copy number signature analysis. **(a)** Copy number alteration signatures identified in the cohort. **(b)** Heatmap showing the hierarchical clustering of samples based on the contributions of Sig1 and Sig2. Comparative analysis with CNA signatures from the Pan-Cancer Analysis of Whole Genomes (PCAWG) revealed a strong similarity between Sig1 and Sig2 with PCAWG CNA signatures CNS7 and CNS14 (cosine similarity > 0.5).

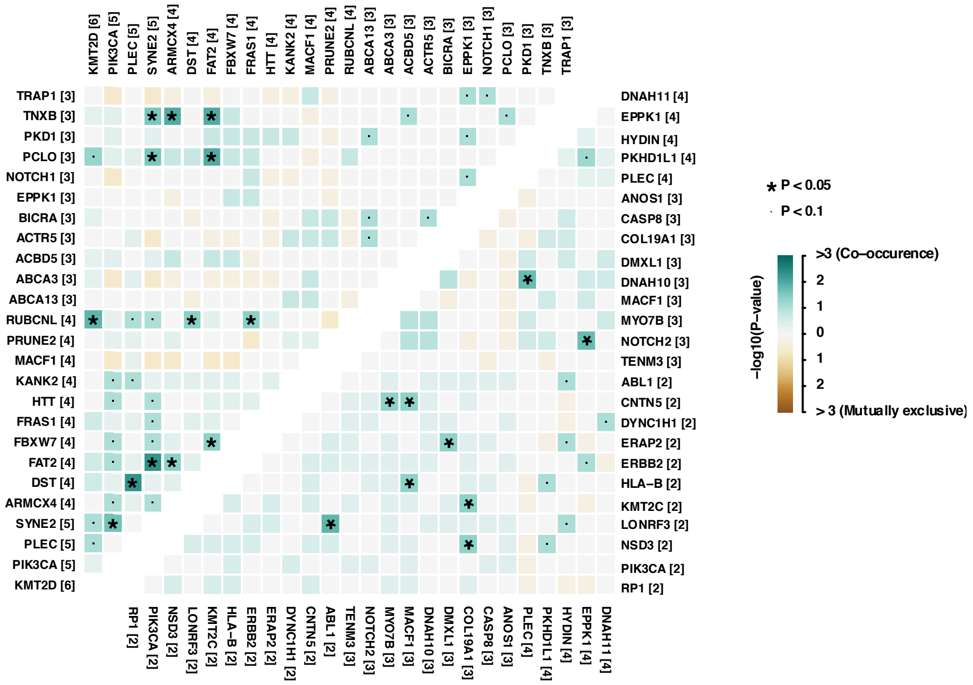

Supplementary Figure 10: Heatmap depicting the co-occurring and mutually exclusive mutated genes across chemoradiation sensitive and resistant cohorts. Mutations in the sensitive cohort are represented in the upper diagonal, while mutations in the resistant cohort are displayed in the lower diagonal.

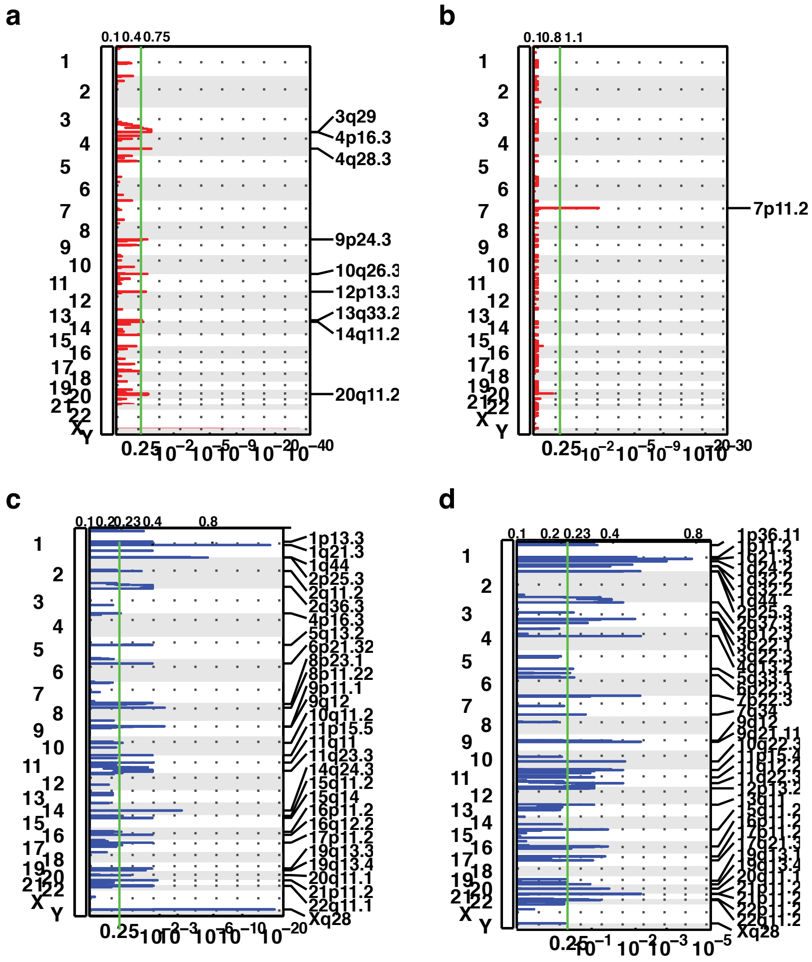

Supplementary Figure 11: Recurrent copy number alterations in chemoradiation sensitive and resistant cervical cancer cohorts. Recurrent **(a)** Amplifications and **(b)** Deletions observed in chemoradiation sensitive and resistant cohorts by GISTIC 2.0. The genome is oriented vertically from top to bottom. GISTIC q-values, indicating the false discovery rate at each locus are presented on a log scale (x axis) and the green threshold line signifies the q-value threshold.

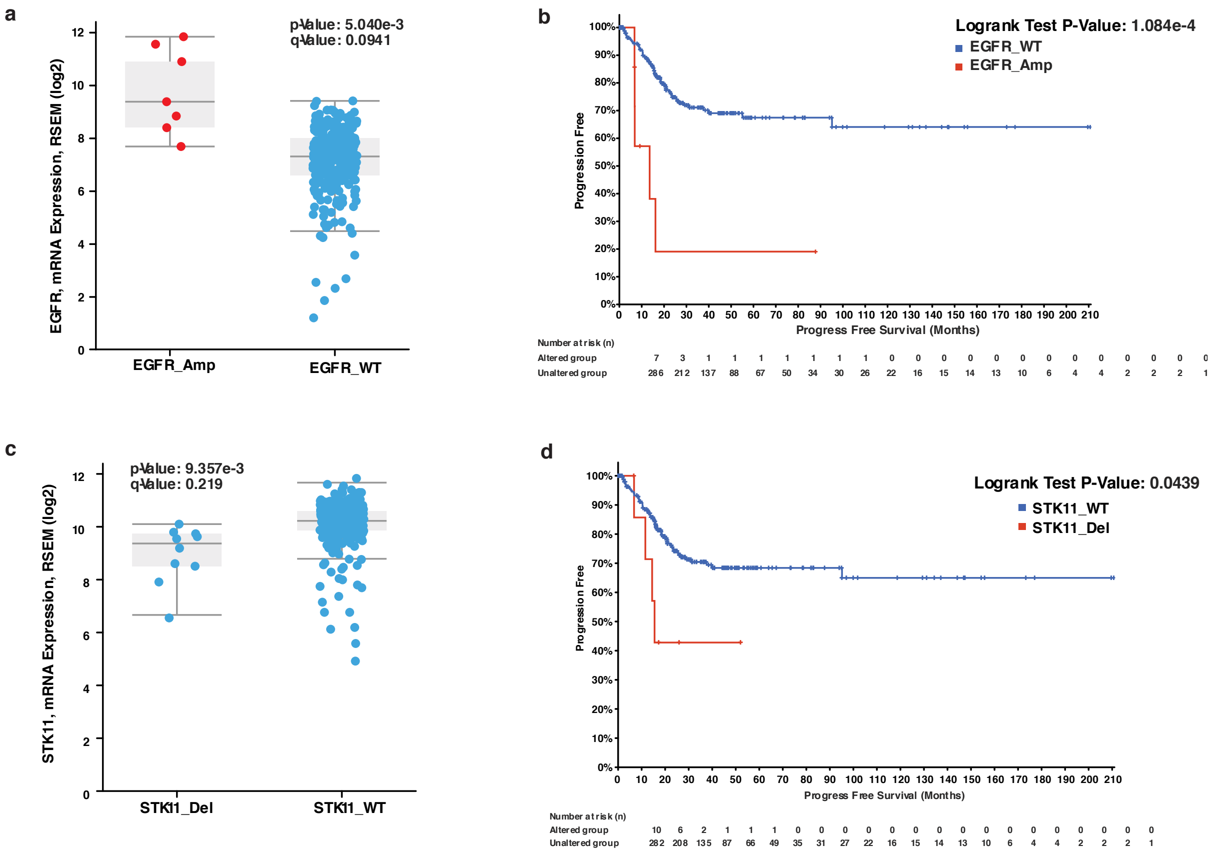

Supplementary Figure 12: Expression and prognostic implications of EGFR amplification and STK11 structural variant deletion in cervical cancer cohort. **(a)** Boxplot comparing RNA expression of EGFR between EGFR amplified group and the wild-type group. The x-axis represents the two groups (altered and wildtype) and y-axis represents the log2 RSEM expression value. **(b)** Kaplan-Meier survival plot illustrating progression-free survival (PFS) in patients with EGFR alterations. The red curve represents patients with EGFR amplification, and the blue curve represents patients with wild-type EGFR. The x-axis indicates progression-free survival in months, and the y-axis represents the percentage of patient's progression-free at each time point. **(c)** Boxplot comparing RNA expression of STK11 between STK11 deletion group and the wild-type group. **(d)** Kaplan-Meier survival plot illustrating progression-free survival (PFS) in patients with STK11 alterations.

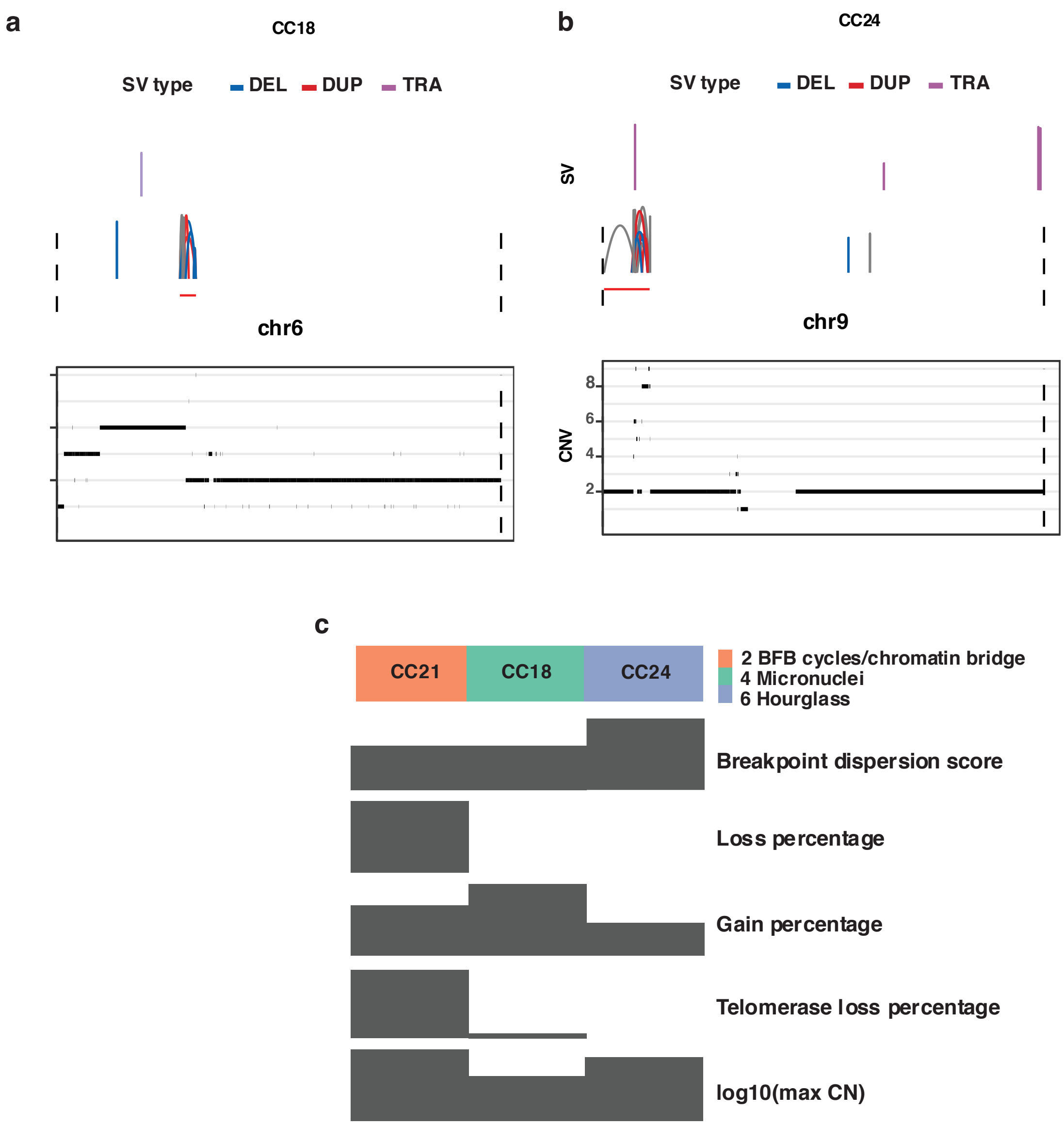

Supplementary Figure 13: Complex genomic rearrangements (CGRs) and signature analysis. **(a) & (b)** SV and CNV profiles of CGRs observed in chromosomes 6 and 9 in two chemoradiation-resistant samples, CC18 and CC24 involving low-level CN gains. **(c)** CGR signatures identified in the samples. Each column represents a unique CGR event, with the top panel displaying the predicted CGR signature for the event. The bottom panels show the normalized values for five distinct features associated with the CGR event, including BFB cycles/chromatin bridges, micronuclei, and the hourglass signature.

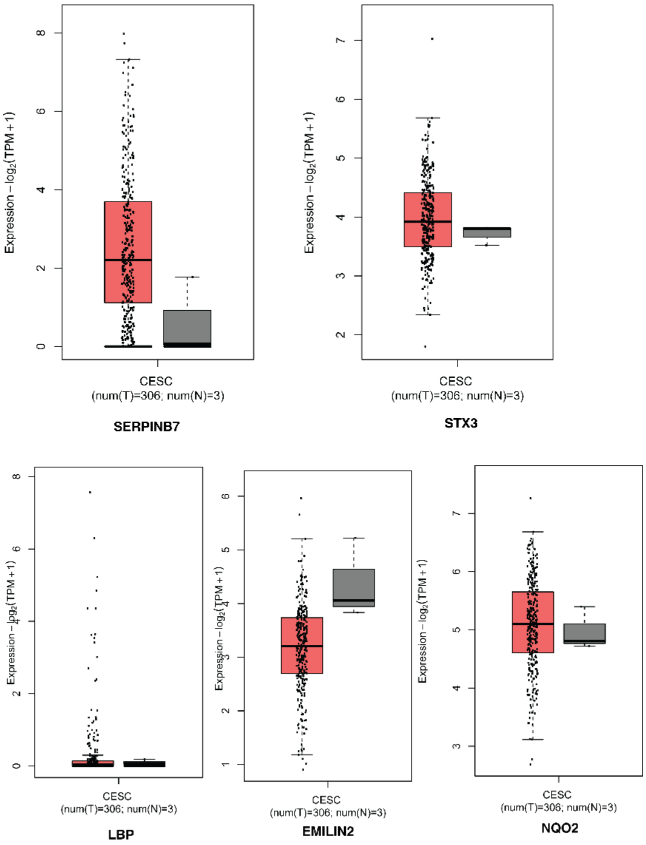

Supplementary Figure 14: The mRNA expression levels of the genes (SERPINB7, STX3, LBP, EMILIN2, and NQO2) in cervical squamous cell carcinoma (CESC) tissues compared to normal tissues. Expression is shown as log2(TPM + 1), with tumor samples (T) in red and normal samples (N) in gray. Sample sizes: tumor (n=306), normal (n=3). Box plots depict median expression levels and interquartile ranges, with individual data points shown as dots

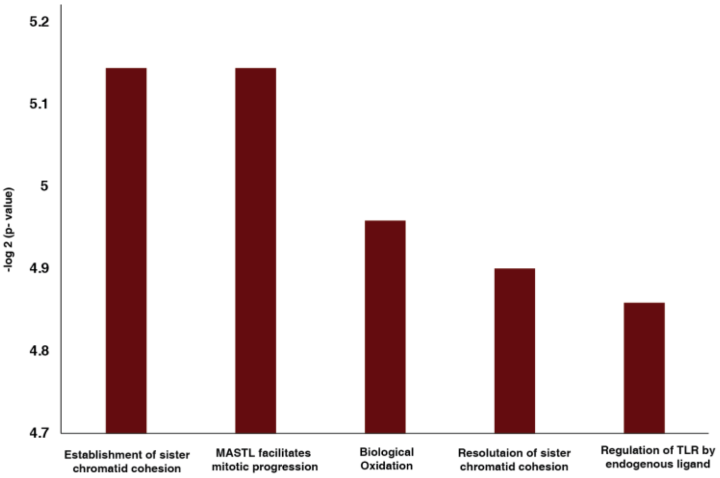

Supplementary Figure 15: Functional enrichment analysis revealed the pathways involved in treatment resistance in cervical cancer patients. Top five pathways enriched in the data set.

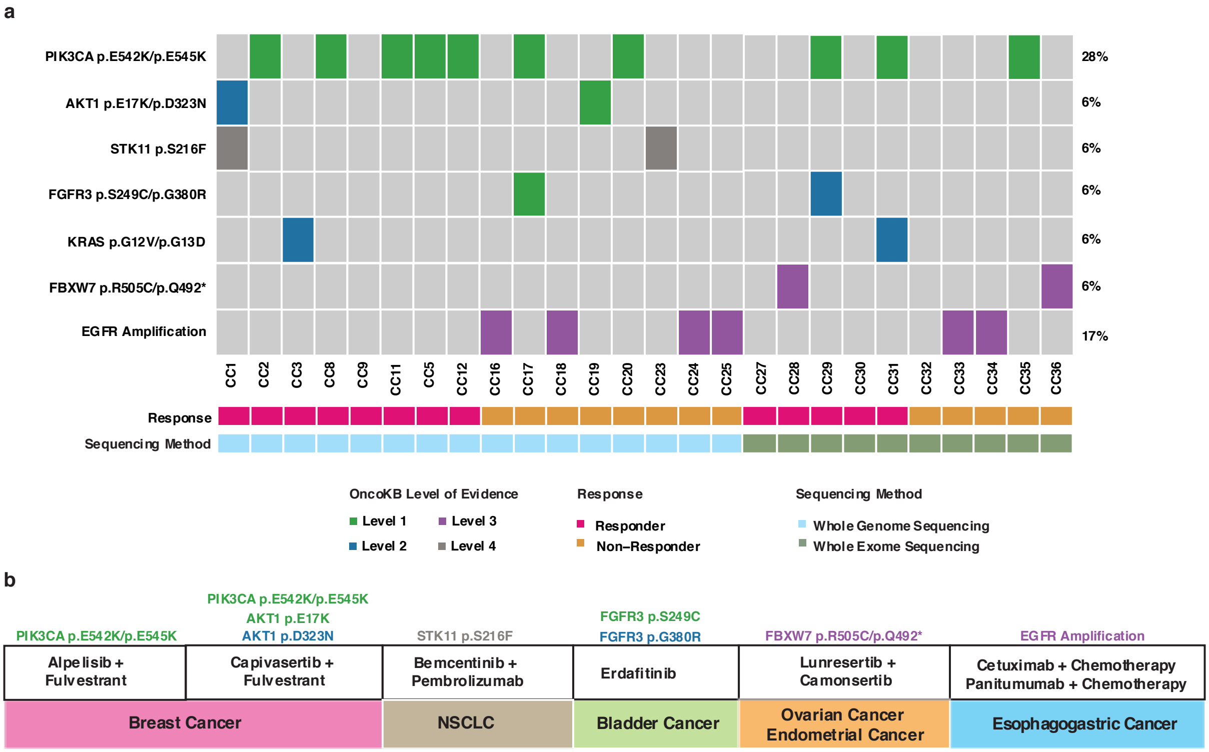

Supplementary Figure 16: Clinically actionable variants and treatments. **(a)** Heatmap depicting the clinically actionable variants identified in each patient. Each column represents a patient; each row represents a gene and its variant. Frequencies displayed on the right axis correspond to the total cohort including WGS and WES. **(b)** The bottom panel outlines FDA-approved or experimental treatments associated with each variant in specific cancers, based on OncoKB evidence.
